# Efficiency enhancement of ruthenium-based DSSCs employing A–π–D–π–A organic Co-sensitizers†

**DOI:** 10.1039/d0ra03916k

**Published:** 2020-07-27

**Authors:** Islam M. Abdellah, Ahmed El-Shafei

**Affiliations:** Department of Chemistry, Faculty of Science, Aswan University Aswan 81528 Egypt islamabdellah2@aswu.edu.eg; Polymer and Color Chemistry Program, North Carolina State University Raleigh 27606 USA Ahmed_El-Shafei@ncsu.edu

## Abstract

A new bipyridyl Ru(ii) sensitizer incorporating triphenylamine and the 3,4-ethylenedioxythiophene (EDOT) ancillary ligand IMA5 was synthesized for dye-sensitized solar cells (DSSCs). The performance of these DSSCs has been enhanced *via* di-anchoring metal-free organic sensitizers, denoted IMA1–4, with structural motif A–π–D–π–A and incorporating phenyl-dibenzothiophene-phenyl (Ph-DBT-Ph) as the main building block but with different anchoring groups (A). These new organic sensitizers were well-characterized and used as efficient co-sensitizers. Their photophysical, electrochemical and photovoltaic properties were studied. Furthermore, molecular modeling studies using DFT calculations were used to investigate their suitability as effective sensitizers/co-sensitizers. The molecular orbital isodensity showed distinguishable delocalization of the intramolecular charge in the DBT moiety. The photovoltaic characterization showed that IMA3 had the best DSSC performance (*η* = 2.41%). In addition, IMA1–4 was co-sensitized in conjunction with the newly synthesized IMA5 complex to enhance light harvesting across expanded spectral regions and thus improve efficiency. The solar cells co-sensitized with IMA2, IMA3 and IMA4 exhibited improved efficiency (*η*) of 6.25, 6.19 and 5.83%, respectively, which outperformed the device employing IMA5 alone (*η* = 5.54%) owing to the improvement in the loading of IMA2, IMA3 and IMA4 in the presence of IMA5 on the surface of the TiO_2_ nanoparticles, and charge recombination was suppressed.

## Abstract 2

The role of metal-free organic sensitizers (IMA1-4) in the improvement of photocurrent efficiency and the overall performance of the DSSCs when used as a co-sensitizer with a Ru(ii) sensitizer (IMA5).

## Graphical abstract

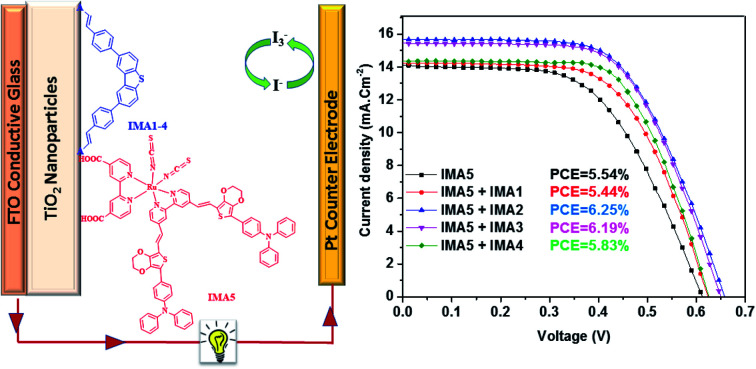

## 1. Introduction

Dye-sensitized solar cells (DSSCs) are one of the third-generation of photovoltaic (PV) technology that represents clean and sustainable energy used to generate electricity from abundant sunlight to tackle the global energy crisis.^1,2^ As promising solar cells, DSSCs have attracted interest from academics and industry because of their low cost, stability, high efficiency and ease of manufacture.^3–6^ DSSCs comprise several different components, such as conductive glass, a mesoporous semiconductor film, electrolytes and sensitizers, so efficient tuning is crucial to realizing the highest efficiency for these major components.^7–11^ In DSSCs, the molecular geometry of the sensitizer should be engineered and designed to achieve broad UV absorption spectra, harmonious thermodynamic properties, suitable molecular orbital energy levels and excellent stability.^12–15^ In addition, the thermodynamic and kinetic properties of the sensitizer are very important for the initiation of light harvesting and for obtaining the electrochemical processes required for effective DSSCs. Thus, systematic methodology is needed to address these issues and thus to design new sensitizers characterized by high-efficiency photoconversion.^16,17^ In this regard, Ru(ii)-based sensitizers have been shown to be viable sensitizers for DSSCs owing to their unusual metal-to-ligand charge transitions (MLCT), unique excited photostability and photophysical properties. The exceptional light harvesting and durability characteristics of these photosensitizers are attributed to the transition (MLCT) through which the photoelectric charge moves to the TiO_2_ faster than the electron recombination with the oxidized dye molecule, instead of moving through the circuit.^18–20^ In addition, metal-free organic sensitizers are the most favored candidates compared to Ru(ii)-based sensitizers because they have many advantages, such as a flexible model, cost-effective synthesis and superior molar extinction coefficients accompanied with intramolecular charge transfer (ICT) from an electron-rich donor to an anchoring unit through a π-spacer unit upon light absorption.^21–24^ In addition to the requirement to improve DSSC performance, co-sensitization is one of the most promising approaches for improving efficiency in DSSCs using a combination of organic sensitizers (visible absorption) and Ru(ii) (NIR-absorbing) complexes in order to obtain a wide spectral response in the visible light region and thus improve the absorption of light.^1,25^ Actually, co-sensitization of DSSCs significantly increases the performance of photovoltaics compared to a single sensitizer.^26–28^ This is attributed to preventing the aggregation of the dye and minimizing charge recombination, increasing the efficient accumulation of both types of dyes on the TiO_2_ surface owing to the difference in the molecular sizes of the dyes, thus facilitating the harvesting of a maximum number of incident photons by the cell.^29^ Different organic push–pull sensitizers have been used in DSSCs as effective co-sensitizers for Ru(ii) complexes compared to individual dye cells; for example, a black dye increased photocurrent efficiency (PCE) by up to 11% when co-sensitized with D131 color dye,^30^ N3 dye when co-sensitized with simple aniline-based D–A architecture enhanced the PCE by up to 7.02%,^1^ Ru sensitizer (SPS-01) co-sensitization with a metal-free dye containing thienylfluorene (JD1) increased the PCE by 8.30% (ref. 31) and a JK2 and SQ01 co-sensitized DSSC showed a 7.43% improvement in efficiency.^32^ Furthermore, triple co-sensitization of Y1 + TP_2_A + HSQ4 improved the PCE by 7.48%.^33^

**Fig. 1:**
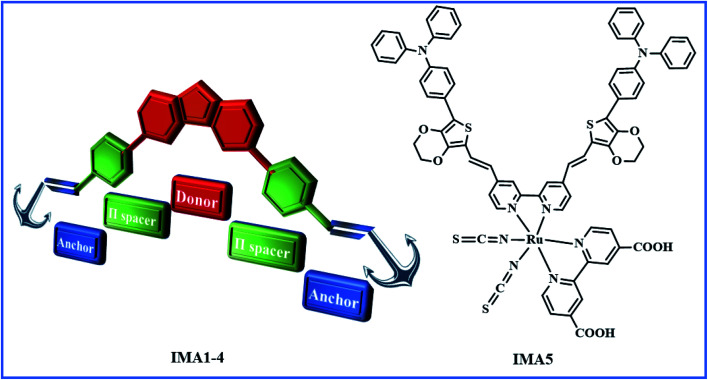
Graphical representation of organic sensitizers IMA1–4 with A–π–D–π–A structure and the IMA5 complex.

**Scheme 1:**
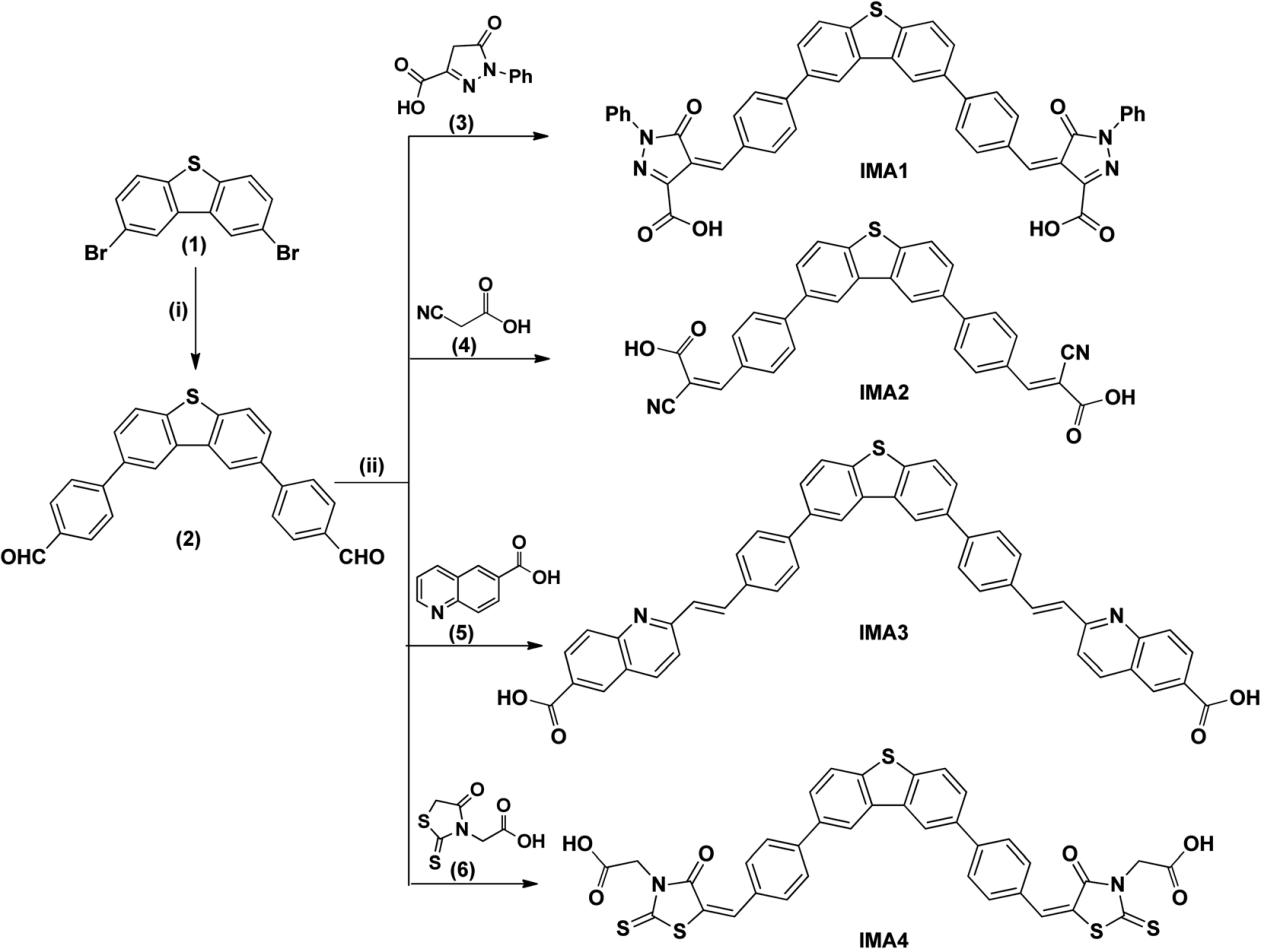
Synthesis of bi-anchoring metal-free organic photosensitizers. (i) *p*-Boronic acid benzaldehyde, K_2_CO_3_, Pd(PPh_3_)_4_, and DMF. (ii) AcONH_4_ and AcOH-glacial.

**Scheme 2:**
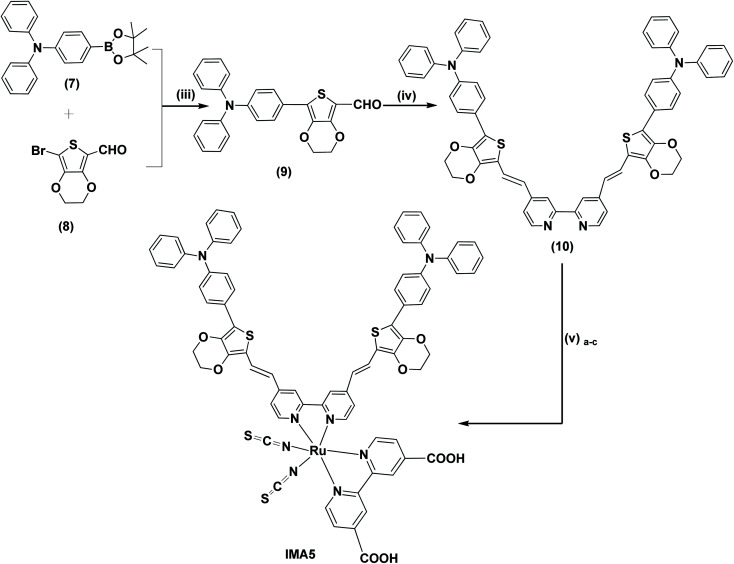
(iii) Aliquat-336, K_2_CO_3_, Pd(PPh_3_)_4_, H_2_O, THF, 90 °C. (iv) 4,4′-Dimethyl-2,2′-dipyridyl, Me_3_SiCl , 115 °C, pressure tube. (v)_a–c_ (a) Dichloro-(*p*-cymene)-ruthenium(ii) dimer, anhydrous DMF, 95 °C, 5 h. (b) 2,2′-Bipyridinyl-4, 4′-dicarboxylic acid, 145 °C, 5 h. (c) NH_4_SCN, 140 °C, 5 h.

Herein, we report the synthesis and characterization of four new organic sensitizers with A–π–D–π–A architecture carrying different acceptor units, *viz*. 1-phenyl-pyrazol-5-one-3-carboxylic acid IMA1, cyanoacetic acid IMA2, 2-methylquinoline-6-carboxylic acid IMA3, rhodamine-3-acetic acid IMA4 and a new ruthenium(ii)-based complex (IMA5) incorporating a bipyridine linked with two branches of a TPA-EDOT molecular motif as the electron donor, and 2,2′-bipyridinyl-4,4′-dicarboxylic acid was used as the main anchoring ligand, as shown in Fig. 1. The synthetic schemes and structures of the new dyes IMA1–5 are depicted in Schemes 1 and 2. The dyes were used as sensitizers/co-sensitizers for the fabrication of DSSCs to evaluate their photovoltaic performance and the interfacial charge recombination process. The target molecules and all compounds were well-characterized using various spectral techniques, such as FT-IR, ^1^H-NMR and high-resolution mass spectroscopy analysis. Their optical bandgap (*E*_0–0_) and electronic energetics (GSOP and ESOP) were measured experimentally using cyclic voltammetry and the orbital charge distributions over the dye molecules were determined utilizing density functional theory (DFT).

## 2. Experimental

### 2.1. Materials and methods

2,8-Dibromodibenzo[*b*,*d*]thiophene, *p*-boronic acid benzaldehyde, pd(dppf)_2_Cl_2_, K_2_CO_3_, Pd(PPh_3_)_4_, EDOT, POCl_3_, rhodamine-3-acetic acid, cyanoacetic acid, 2-methyl quinoline-6-carboxylic acid and 5-oxo-1-phenyl-pyrazole-3-carboxylic acid were purchased from Sigma-Aldrich, Alfa Aesar and Ark Pharm. Furthermore, 2,2′-bipyridinyl-4,4′-dicarboxylic acid,^34^*N*,*N*-diphenyl-4-(4,4,5,5-tetramethyl-1,3,2-dioxaborolan-2-yl)aniline (7)^24^ and 2-bromo-(3,4-ethylenedioxythiophene)-5-carbaldehyde (8)^35^ were synthesized as reported with the detailed procedures included in the ESI.† All solvents were purchased from Fisher Scientific. ^1^H-NMR spectra were recorded utilizing a Bruker AVANCE 500 MHZ using DMSO-*d*_*6*_ as the solvent and tetramethylsilane for calibrating the chemical shift. The mass spectra were recorded using a high-resolution Thermo Scientific Exactive Plus. The FTIR spectra were recorded for the pure solid using a Bruker ALPHA spectrophotometer. The UV-vis spectra were recorded in 1 × 10^−5^ M solutions in an appropriate solvent using a Varian Cary 3 UV-vis spectrophotometer. Cyclic voltammetry (CV) was carried out for all sensitizers in an appropriate anhydrous solution at a scan rate of 50 mV s^−1^ with 0.1 M tetra-*n*-butylammonium hexafluorophosphate (*n*-Bu)_4_N^+^(PF_6_)^−^ as the supporting electrolyte at room temperature using a Vertex electrochemical workstation. The photovoltaic parameters of the DSSCs were measured in an Oriel SOL3A class AAA solar simulator with an AM 1.5G spectral filter. A QEX10 measurement system was used to run IPCE experiments. The electrochemical impedance spectra, including Nyquist and Bode curves, were obtained using a Biologic SP-150 with a AAA solar simulator. Molecular modeling calculations (DFT) were carried out using the Gaussian 09 software package and the calculations were performed remotely at the NC State University High Performance Computing (HPC).

### 2.2. Synthesis and characterization

The synthetic routes for the metal-free organic dyes with A–π–D–π–A architecture (IMA1–4) along with ruthenium complex IMA5 are presented in Schemes 1 and 2. The synthesis starts with the C–C Suzuki coupling reaction of 2,8-dibromodibenzo[*b*,*d*]thiophene (1) with two moles of *p*-boronic acid benzaldehyde in potassium carbonate solution and tetrakis(triphenylphosphine)palladium catalyst to afford 4,4′-(dibenzo[*b*,*d*]thiophene-2,8-diyl)dibenzaldehyde (2). The dialdehyde (2) then undergoes a Knoevenagel condensation reaction with active methylene compounds, such as rhodamine-3-acetic acid (3), cyanoacetic acid (4), 2-methyl quinoline-6-carboxylic acid (5) and 1-phenyl-pyrazol-5-one-3-carboxylic acid (6), in the presence of ammonium acetate catalyst to give the target organic sensitizers IMA1–4. Synthesis of the target Ru complex starts with the C–C Suzuki cross-coupling reaction of *N*,*N*-diphenyl-4-(4,4,5,5-tetramethyl-1,3,2-dioxaborolan-2-yl)aniline (7)^24^ and 2-bromo-(3,4-ethylenedioxythiophene)-5-carbaldehyde (8)^35^ to prepare the required aldehyde (9). The aldehyde (9) undergoes a condensation reaction with 4,4′-dimethyl-2,2′-dipyridyl in the presence of Me_3_SiCl to afford the ancillary ligand (10). Finally, the target Ru(ii) complex IMA5 is synthesized *via* a one-pot three-step reaction protocol, wherein the precursor ancillary ligand (10) is reacted with dichloro-(*p*-cymene)-ruthenium(ii) dimer followed by 2,2′-bipyridyl-4,4′-dicarboxylic acid and ammonium thiocyanate. All the new compounds and photosensitizers were purified using column chromatography using an appropriate eluent and their synthesis was confirmed with various spectral techniques, as described in the ESI (Fig. S4–S27).† More details on the synthetic procedures for IMA1–5 are provided in the following sections.

#### 2.2.1. Synthesis of 4,4′-(dibenzo[*b*,*d*]thiophene-2,8-diyl)dibenzaldehyde (2)

A DMF solution of 2,8-dibromodibenzo[*b*,*d*]thiophene (1) (0.342 g, 0.001 mmol), *p*-boronic acid benzaldehyde (0.329 g, 0.00219 mmol) and potassium carbonate solution (0.691 g, 0.00499 mmol) were added to a three-necked flask. The solution was purged with argon for 30 min and then tetrakis(triphenylphosphine)palladium (0.034 g, 0.03 mmol) was added. The reaction mixture was stirred at 80 °C overnight. The reaction was followed by TLC until reaction completion, then the reaction mixture was left to cool down and quenched by adding 50 mL of water, followed by extraction with ethyl acetate (3 × 30 mL). The organic layer was dried using anhydrous Mg_2_SO_4_ and the solvent was removed under vacuum. The main product was purified by silica column chromatography with a mixture of hexane and ethyl acetate (3 : 1). The compound crystallized from ethanol and gave a pure white powder. HRMS-ESI (*m*/*z*): [M + H]^+^ calcd. for C_26_H_17_O_2_S: 393.09438; found: 393.09408 (error, Δ*M*: −0.767 ppm). FT-IR (cm^−1^): 3015 (=CH alkene), 1707 (C=O), 1583 (C=C aromatic conjugation), 860–680 (aromatic CH bending). ^1^H NMR (500 MHz, DMSO-*d*_6_) δ: 9.85 (s, 2H), 7.72–7.65 (m, 2H), 7.40–7.32 (m, 4H), 7.16–7.05 (m, 6H), 7.02–6.95 (m, 2H).

#### 2.2.2. Synthesis of 4,4′-((dibenzo[*b*,*d*]thiophene-2,8-diylbis(4,1-phenylene))bis (methanylylidene))bis(5-oxo-1-phenyl-4,5-dihydro-1*H*-pyrazole-3-carboxylic acid) (IMA1)

A mixture of 4,4′-(dibenzo[*b*,*d*]thiophene-2,8-diyl)dibenzaldehyde (2) (0.393 g, 1 mmol) and 1-phenyl-pyrazol-5-one-3-carboxylic acid (3) (0.4 g, 2 mmol) in 30 mL of glacial acetic acid was refluxed in the presence of ammonium acetate (0.3 g, 3.9 mmol) for 6 hours under an argon atmosphere at 118 °C. Upon completion of the reaction, the reaction mixture was cooled to room temperature and poured into ice-cold water to provide an orange precipitate. The precipitate was filtered and purified by column chromatography using silica gel and CHCl_3_ : CH_3_OH (10 : 3) as the mobile phase to obtain a yellow-orange solid, which was crystallized from hexane. HRMS-ESI (*m*/*z*): [M + H]^+^ calcd for C_46_H_29_N_4_O_6_S: 765.18133; found: 765.18394 (error, Δ*M*: 3.419 ppm). FT-IR (cm^−1^): 3374 (OH carboxylic), 3026 (=CH alkene), 1706 (C=O), 1596 (C=C aromatic conjugation), 860–680 (aromatic CH bending). ^1^H NMR (500 MHz, DMSO-*d*_6_) *δ*: 9.95 (s, 2H), 8.12–8.07 (m, 2H), 8.03 (dd, *J* = 1.8, 0.6 Hz, 2H), 7.99–7.94 (m, 4H), 7.92–7.84 (m, 2H), 7.76–7.53 (m, 4H), 7.39–7.34 (m, 4H), 7.16–7.02 (m, 8H).

#### 2.2.3. Synthesis of 3,3′-(dibenzo[*b*,*d*]thiophene-2,8-diylbis(4,1-phenylene))bis(2-cyanoacrylic acid) (IMA2)

A mixture of 4,4′-(dibenzo[*b*,*d*]thiophene-2,8-diyl)dibenzaldehyde (2) (0.393 g, 1 mmol) and cyanoacetic acid (4) (0.212 g, 2.5 mmol) in 30 mL of glacial acetic acid was refluxed in the presence of ammonium acetate (0.3 g, 3.9 mmol) for 6 hours under an argon atmosphere at 118 °C. Upon completion of the reaction, the reaction mixture was cooled to room temperature and poured into ice-cold water to provide a yellowish precipitate. The precipitate was filtered and purified by column chromatography using silica gel and CHCl_3_ : CH_3_OH (10 : 3) as the mobile phase to obtain a yellow color solid, which was crystallized from hexane. HRMS-ESI (*m*/*z*): [M − H]^−^ calcd for C_32_H_17_N_2_O_4_S: 525.09145; found: 525.09246 (error, Δ*M*: 4.012 ppm). FT-IR (cm^−1^): 3331 (OH carboxylic), 3033 (=CH alkene), 2256 (CN), 1703 (C=O), 1590 (C=C aromatic conjugation), 860–680 (aromatic CH bending). ^1^H NMR (500 MHz, DMSO-*d*_6_) *δ*: 8.92 (s, 2H), 8.85 (d, *J* = 1.7 Hz, 2H), 8.46–8.39 (m, 4H), 8.36 (dd, *J* = 8.8, 1.8 Hz, 2H), 7.89–7.86 (m, 2H), 7.79–7.63 (m, 4H), 7.56–7.53 (m, 2H).

#### 2.2.4. Synthesis of 2,2′-((dibenzo[*b*,*d*]thiophene-2,8-diylbis(4,1-phenylene))bis(ethene-2,1-diyl))bis(quinoline-6-carboxylic acid) (IMA3)

A mixture of 4,4′-(dibenzo[*b*,*d*]thiophene-2,8-diyl)dibenzaldehyde (2) (0.393 g, 1 mmol) and 2-methyl-quinoline-6-carboxylic acid (5) (0.411 g, 2.2 mmol) in 30 mL of glacial acetic acid was refluxed in the presence of ammonium acetate (0.3 g, 3.9 mmol) for 6 hours under an argon atmosphere at 118 °C. After the reaction completion, the reaction mixture was cooled to room temperature and poured into ice-cold water to afford a reddish precipitate. The precipitate was filtered and purified by column chromatography using silica gel and CHCl_3_ : CH_3_OH (10 : 3) as the mobile phase to obtain a red solid, which was crystallized from hexane. HRMS-ESI (*m*/*z*): [M]^+^ calcd for C_48_H_30_N_2_O_4_S: 730.66641; found: 730.66785 (error, Δ*M*: 3.031 ppm). FT-IR (cm^−1^): 3396 (OH carboxylic), 3070 (=CH alkene), 1702 (C=O), 1595 (C=C aromatic conjugation), 860–680 (aromatic CH bending). ^1^H NMR (500 MHz, DMSO-*d*_6_) *δ*: 9.94 (s, 2H), 8.10–8.06 (m, 2H), 8.01 (d, *J* = 1.8 Hz, 1H), 7.96–7.93 (m, 4H), 7.89 (d, *J* = 1.3 Hz, 1H), 7.72 (d, *J* = 8.6 Hz, 2H), 7.39–7.32 (m, 7H), 7.14–7.07 (m, 7H), 7.05–7.03 (m, 4H).

#### 2.2.5. Synthesis of 2,2′-((dibenzo[*b*,*d*]thiophene-2,8-diylbis(4,1-phenylene))bis (methanylylidene))bis(4-oxo-2-thioxothiazolidin-3-yl-5-ylidene))diacetic acid (IMA4)

A mixture of 4,4′-(dibenzo[*b*,*d*]thiophene-2,8-diyl)dibenzaldehyde (2) (0.393 g, 1 mmol) and rhodamine-3-acetic acid (6) (0.478 g, 2.5 mmol) in 30 mL of glacial acetic acid was refluxed in the presence of ammonium acetate (0.3 g, 3.9 mmol) for 18 hours under an argon atmosphere at 118 °C. Upon completion of the reaction, the reaction mixture was cooled to room temperature and poured into ice-cold water to provide a yellowish precipitate. The precipitate was filtered and purified by silica gel column chromatography using CHCl_3_ : CH_3_OH (10 : 3) as the mobile phase to obtain dark yellow solid, which was crystallized from hexane. HRMS-ESI (*m*/*z*): [M − H]^−^ calcd for C_36_H_22_N_2_O_6_S_5_: 737.00086; found: 737.00141 (error, Δ*M*: 0.745 ppm). FT-IR (cm^−1^): 3337 (OH carboxylic), 1649 (C=O), 1517 (C=C aromatic conjugation), 860–680 (aromatic CH bending). ^1^H NMR (600 MHz, DMSO-*d*_*6*_) *δ*: 9.06 (s, 2H), 8.63 (d, *J* = 1.8 Hz, 2H), 8.54 (d, *J* = 1.7 Hz, 2H), 8.05 (d, *J* = 20.0 Hz, 2H), 7.87–7.79 (m, 4H), 7.76 (dd, *J* = 8.6, 2.9 Hz, 1H), 7.72–7.65 (m, 4H), 7.62–7.51 (m, 1H), 4.75 (s, 4H).

#### 2.2.6. Synthesis of 7-(4-(diphenylamino)phenyl)-2,3-dihydrothieno[3,4-*b*][1,4]dioxine-5-carbaldehyde (9)

In a three-necked flask a mixture of *N*,*N*-diphenyl-4-(4,4,5,5-tetramethyl-1,3,2-dioxaborolan-2-yl)aniline (7) (0.408 g, 1.1 mmol), 2-bromo-(3,4-ethylenedioxythiophene)-5-carbaldehyde (8) (0.249 g, 1 mmol) and tetrakis(triphenylphosphine)palladium (0.034 g, 0.03 mmol) was dissolved in THF and degassed under an argon atmosphere for 15 minutes then a K_2_CO_3_ (2.5 mL, 2 M) solution was added. The reaction mixture was stirred at 80 °C for 5–6 hours and followed by TLC until completion. After the reaction completion, it was left to cool down and quenched by adding (50 mL) of water then extracted with CH_2_Cl_2_ (3 × 40 mL). The organic layer was dried using anhydrous Mg_2_SO_4_ and the organic solvent was removed under vacuum. The crude product was purified by column chromatography on silica with CHCl_3_. The compound was crystallized from hexane and give a yellow crystal. HRMS-ESI (*m*/*z*): [M + H]^+^ calcd for C_25_H_19_NO_3_S: 414.11584; found: 414.11557 (error, Δ*M*: −0.643 ppm). FT-IR (cm^−1^): 3032 (=CH alkene), 2803 (CH for CH_2_) 1637 (C=O), 1519 (C=C aromatic conjugation), 860–680 (aromatic CH bending). ^1^H NMR (500 MHz, DMSO-*d*_*6*_) *δ*: 9.80 (s, 1H), 7.82–7.6 (m, 2H), 7.64–7.55 (m, 2H), 7.26 (t, *J* = 7.5 Hz, 4H), 7.11–6.90 (m, 4H), 7.21 (tt, *J* = 7.4, 2.0 Hz, 2H), 4.37 (s, 4H).

#### 2.2.7. Synthesis of 4,4′-(([2,2′-bipyridine]-4,4′-diylbis(ethene-2,1-diyl))bis(2,3-dihydrothieno[3,4-*b*][1,4]dioxine-7,5-diyl))bis(*N*,*N*-diphenylaniline) (10)

The ancillary ligand (10) was synthesized under pressure in a glass tube containing 4,4′-dimethyl-2,2′-bipyridine (0.184 g, 1 mmol), 7-(4-(diphenylamino)phenyl)-2,3-dihydrothieno[3,4-*b*][1,4]dioxine-5-carbaldehyde (9) (0.8269 g, 2 mmol), 1.52 mL of chlorotrimethylsilane (12 mmol), and 50 mL of anhydrous DMF. The tube was well closed by the cap and heated at 100 °C in an oil bath for 48 hours with continuous stirring. Over the course of 48 hours, the color of the reaction mixture changed from yellow to dark orange. At the end of the reaction, the pressure was released after the tube was cooled and the solvent was removed using a rotary evaporator; a dark orange liquid was deposited with the addition of 50 mL of ice and water. Finally, vacuum filtration was performed to supply the well-washed antenna ligand. The antenna ligand was then dried overnight at 50 °C giving a 68% yield. The antenna ligand was recrystallized from acetone to form pure dark brown crystals. HRMS-ESI (*m*/*z*): [M + H]^+^ calcd for C_62_H_46_N_4_O_4_S_2_: 975.30332; found: 975.30276 (error, Δ*M*: −0.578 ppm). FT-IR (cm^−1^): 3033 (=CH), 1587 (C=C aromatic conjugation), 860–680 (aromatic CH bending). ^1^H NMR (500 MHz, DMSO-*d*_6_) *δ*: 8.67–8.63 (m, 2H), 8.61–8.57 (m, 2H), 7.67–7.59 (m, 4H), 7.36 (d, *J* = 2.0 Hz, 2H), 7.34–7.27 (m, 12H), 7.10 (dd, *J* = 2.7, 1.3 Hz, 2H), 7.07–6.99 (m, 14H), 4.43 (s, 8H).

#### 2.2.8. Synthesis of ruthenium(ii) complex (IMA5)

The synthesis of IMA5 was carried out in a single-pot three-step reaction. The reactions were carried out under argon gas in a 100 mL flask connected with a condenser. The flask was charged with anhydrous DMF, dichloro-(*p*-cymene)-ruthenium(ii) dimer (0.3 g, 0.5 mmol) and ancillary ligand (10) (0.975 g, 1 mmol). The reaction mixture was stirred at 95 °C for 5 h. Then, 2,2′-bipyridyl-4,4′-dicarboxylic acid was added (0.244 g, 1 mmol) and the temperature was raised to 145 °C and the reaction was allowed to run for 6 hours. After the 6 hours, an excess of NH_4_NCS (0.5 g) was added to the reaction mixture and then the reaction was allowed to run for an additional 4 hours at 140 °C. The product was cooled to 25 °C and transferred to a 250 mL round bottom flask and then the DMF was evaporated using a rotary evaporator. Ice was added to the flask and the insoluble precipitate was filtered and washed with deionized H_2_O and ether. Upon drying, the dye was dissolved in CH_3_OH with the addition of 2 mL of tetrabutylammonium hydroxide (TBAOH) and then purified on a silica gel column. The main band (violet) was collected and acidified using 0.1 M HCl to reduce the pH to 2.0 and then allowed to precipitate for 48 hours at low temperature. The precipitate was then filtered and washed with plenty of deionized water to bring the pH to neutral. The pure dye was then dried overnight and collected as dark crystals (yield 74%). HRMS-ESI (*m*/*z*): [M + H]^+^ calcd for C_76_H_55_N_8_O_8_S_4_Ru: 1437.20637; found: 1437.20761 (error: Δ*M*, 0.86184 ppm). FT-IR (cm^−1^): 3388 (OH carboxylic), 3060 (=CH alkene), 2932 (CH for CH_2_) 2104 (SCN), 1718 (C=O), 1589 (C=C aromatic conjugation), 860–680 (aromatic CH bending). ^1^H NMR (500 MHz, DMSO-*d*_6_) *δ*: 8.77 (S, 2H), 8.16–8.00 (m, 4H), 7.88–7.78 (m, 4H), 7.58 (d, *J* = 7.5 Hz, 2H), 7.29–7.16 (m, 14H), 7.07 (d, *J* = 1.5 Hz, 2H), 7.04–6.95 (m, 14H), 6.92 (dd, *J* = 7.4, 1.5 Hz, 2H), 6.89 (dd, *J* = 7.5, 1.4 Hz, 2H), 4.36 (s, 8H).

## 3. Results and discussion

### 3.1. Photophysical and electrochemical properties

**Fig. 2:**
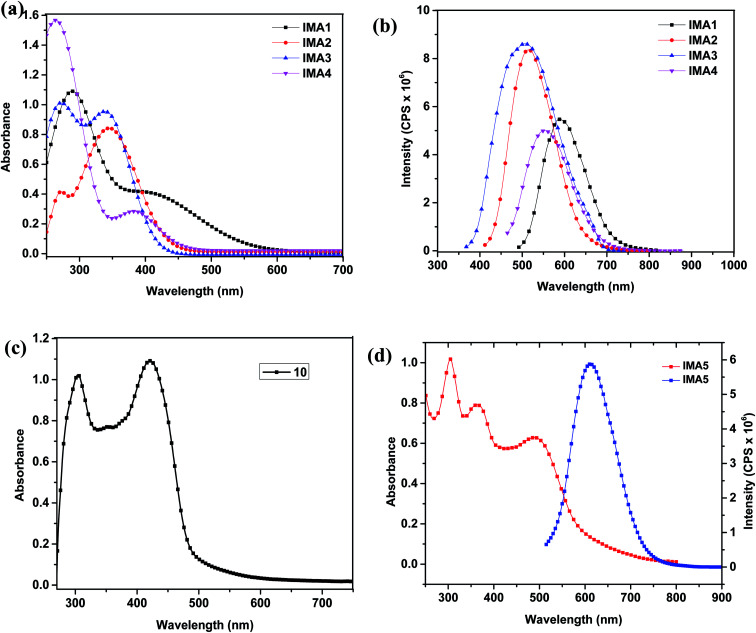
(a) UV-vis absorption spectra and (b) emission spectra of 1 × 10^−5^ M IMA1–4 in DMSO. (c) UV-vis absorption spectrum of 1 × 10^−5^ M ancillary ligand (10) in CHCl_3_. (d) UV-vis absorption spectra of 1 × 10^−5^ M IMA5 in CHCl_3_.

The UV-vis absorption/emission spectra of IMA1–4 were measured in DMSO solution with a concentration of 1 × 10^−5^ M, as displayed in Fig. 2a and b, and their characteristic spectral data are tabulated in Table 1. The lower wavelength bands around 250–300 nm in the absorption spectra of IMA1–4 can be assigned to π → π* transitions localized within the phenyl-DBT-phenyl (π–D–π) moieties. Moreover, the bathochromic shift bands in the region of 350–430 nm can be accredited to intramolecular charge transfer (ICT) from the donor (DBT) to the anchoring moiety *via* the conjugated phenyl segment, which is critical to absorbing lower energy light with reasonable molar absorptivity.^36^ Although the maximum wavelengths (ICT) for photosensitizers IMA1 (λ_max_ = 411 nm) and IMA4 (λ_max_ = 383 nm) are bathochromically shifted compared to those of IMA2 (λ_max_ = 347 nm) and IMA3 (λ_max_ = 339 nm), the molar extinction coefficients of 0.40 × 10^5^ M^−1^ cm^−1^ and 0.29 × 10^5^ M^−1^ cm^−1^ for IMA1 and IMA4, respectively, are extremely low compared to those for IMA2 (*ε*_max_ = 0.85 × 10^5^ M^−1^ cm^−1^) and IMA3 (*ε*_max_ = 0.95 × 10^5^ M^−1^ cm^−1^). It is well known that the capacity to harvest light increases for photosensitizers characterized by higher molar extinction coefficients.^37,38^ On the other hand, the UV-vis absorption/emission spectrum of IMA5 was measured in 1 × 10^−5^ M CHCl_3_ solution and its spectral behavior is depicted in Fig. 2d and the corresponding data are outlined in Table 1. Fig. 2d shows that IMA5 possesses three distinctive absorption bands. The band in the region of 290–320 nm is attributed to the π–π* electronic transition of the bipyridine ligand, while the peak in the 360–400 nm region can be ascribed to ligand-to-ligand charge transfer (LLCT) mixed with metal-to-ligand charge transfer (MLCT) (πd–π*) and the broad peak at 480–525 nm corresponding to the longest wavelength can be credited to metal-to-ligand charge transfer (MLCT) (πd–π*), which corresponds to electron transfer from the HOMO to LUMO energy levels. This is confirmed by comparing the absorption spectrum of the ancillary ligand (10), which is characterized by the presence of π–π* and (LLCT) peaks only, as shown in Fig. 2c, with that of the IMA5 complex, which is characterized by the presence of one more peak for MLCT. The IMA5 complex is characterized by a molar extinction coefficient (*ε*_max_) of 0.624 × 10^5^ M^−1^ cm^−1^). The higher extinction coefficient of the IMA5 complex in the visible region is attributed to the presence of the strong electron donor part, which comprises TPA-EDOT and is directly connected to the bipyridine to form a ligand that contains extended π conjugation, and the directionality of the excited state by perfect tuning of the ligand LUMO energy level with the donating groups. Moreover, the emission spectra of the metal-free organic sensitizers displayed a single emission band in the range of 500–600 nm and the maximum emission wavelength (λ_emi_) is in the order of IMA1 (578 nm) > IMA4 (558 nm) > IMA2 (520 nm) > IMA3 (503 nm), while the IMA5 complex displays a characteristic λ_emi_ in the longer wavelength region of 600–650 nm. Furthermore, the wavelength of the intersection (*I*) of the absorption and fluorescence spectra gives the optical bandgap (*E*_0–0_), which is calculated by converting the wavelength of the intersection (I) from nm to eV. The optical band gaps are in the following order: IMA3 (3.09 eV) > IMA2 (2.86 eV) > IMA4 (2.70 eV) > IMA1(2.57 eV) > IMA5 (2.22 eV) and the Stokes shifts are: IMA1 (167 nm), IMA2 (173 nm), IMA3 (164 nm), IMA4 (175 nm), and IMA5 (110 nm).

**Table 1:** Photophysical and electrochemical data for the synthesized sensitizers

Dye	*λ*_max_ (nm)	*ε*_max_ (10^5^ M^−1^ cm^−1^)	Stokes shift (nm)	*I* (nm)	*E*_max_ (nm)	*E*_0–0_ (eV)	*E* ^Oxd^_Onset_	GSOP (eV)	ESOP (eV)	*ΔG*inj° (eV)
IMA1	411 (ICT)	0.401	167	482	578	2.57	0.28	−5.54	−2.97	1.23
IMA2	347 (ICT)	0.848	173	433	520	2.86	0.24	−5.50	−2.64	1.56
IMA3	339 (ICT)	0.953	164	401	503	3.09	0.20	−5.46	−2.37	1.83
IMA4	383 (ICT)	0.291	175	458	558	2.70	0.21	−5.47	−2.77	1.43
IMA5	505 (d–π*)	0.624	110	557	615	2.22	0.31	−5.57	−3.35	0.85

Cyclic voltammetry (CV) of IMA1–5 is critical for exploring the electronic processes at the mesoporous TiO_2_/dye/electrolyte interface and showing the required energy levels for electron injection to the conduction band (CB) of TiO_2_ and regeneration of the oxidized dyes.^39^ The measurements were performed using a Vertex electrochemical instrument with a three-electrode cell comprising a glassy carbon working electrode where the oxidation or reduction takes place, a platinum disc as the counter electrode, and an Ag/AgCl reference electrode. The obtained voltammograms were used to calculate the ground state oxidation potential (GSOP) from the first jump in the voltammogram, which represents the onset oxidation potential (*E*^Oxd^_Onset_), as shown in the ESI (Fig. S1 and S2).† The onset potential values were calibrated by cyclic voltammetry measurement of ferrocene (Fc/Fc^+^), as shown in the ESI (Fig. S3).† The resulting onset oxidation potential was converted to NHE by applying the following equation: GSOP/NHE = oxidation onset − GSOP/Fc + 0.63. The calculated GSOP/NHE was then converted to electron volts by applying the following equation: GSOP/eV = GSOP/NHE + 4.7. On the other hand, the excited state oxidation potential (ESOP) was calculated from the GSOP value and the calculated energy bandgap (*E*_0–0_) by applying the following equation: ESOP = [GSOP − *E*_0−0_]. The calculated data are tabulated in Table 1. From the results, it is obvious that all the dyes display GSOP energy levels below the CB level of TiO_2_ (−4.2 eV)^40^ and far away from the electrolyte potential (−5.2 eV),^41^ which enables the dye regeneration process. On the other hand, the ESOP levels are energetically higher than that of the CB potential of TiO_2_ to permit electron injection from the excited dye molecules and decrease the recombination process.

**Fig. 3:**
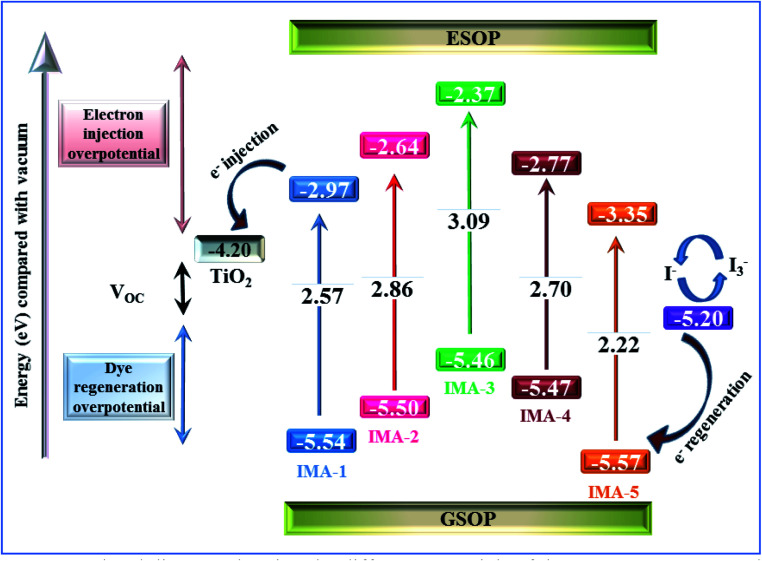
Energy level diagram showing the different potentials of the DSSC components along with the GSOP and ESOP of the sensitizers IMA1–5.

A comparison of the electronic energy levels of the dyes IMA1–5 and the TiO_2_ and *I*^−^/*I*_3_^−^ redox couple energy levels is summarized in Fig. 3. The energy diagram shows that all of the dyes are thermodynamically favorable for electron injection and dye regeneration in the fabricated devices.

### 3.2. Theoretical studies

**Fig. 4:**
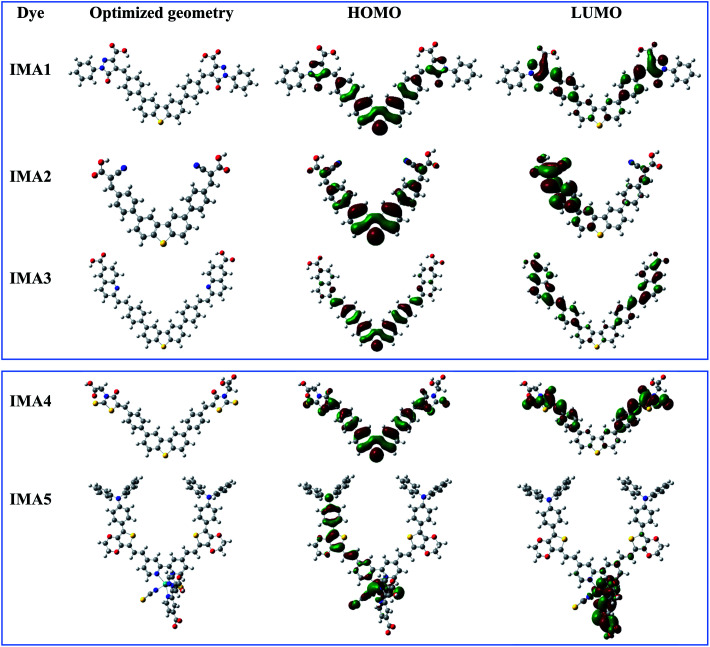
Optimized geometry and HOMO–LUMO molecular orbitals for sensitizers IMA1–5.

Theoretical studies (quantum calculations) were performed on sensitizers IMA1–5 utilizing DFT to understand their geometrical electronic distribution and evaluate their photophysical properties for use in DSSCs. All calculations were performed through North Carolina State University's High-Performance Computing utilizing GAUSSIAN 09 software. The ground state geometries were optimized using the B3LYP energy functional and the DGTZVP basis set. The solvation effect was taken into consideration utilizing the CPCM model in DMSO for dyes IMA1–4 and CHCl_3_ for dye IMA-5. The resulting 3D optimized structures of IMA1–5 with the isosurfaces of the HOMO–LUMO frontier molecular orbitals are presented in Fig. 4. It was obvious that all sensitizers achieved good orbital distribution between the HOMO and LUMO isosurfaces. In the case of the metal-free organic sensitizers IMA1–4, the HOMOs are accumulated on the DBT moiety, which represents the donor part, while the LUMOs are distributed on the acceptor parts, namely the pyrazole carboxylic acid, cyanoacetic acid, quinoline carboxylic acid and thiazole carboxylic acid moieties. On the other hand, in the Ru(ii) sensitizer IMA-5 the HOMO was located on the donor (TPA-EDOT) moiety and the LUMO on the acceptor part, which represents dibipyridine dicarboxylic acid.

The optimized geometries for dyes IMA1–4 have maximum lengths of 28 599 Å, 17 760 Å, 23 095 Å, and 23 558 Å, respectively. The small dyes lengths and its planar structures can make these dyes a strong co-sensitizer for the IMA5 octahedral complex. All of these conditions make it easy for IMA1–4 to fill the gaps left by the bulky IMA5 complex on the TiO_2_ surface. It results in more dye packing on the TiO_2_ surface, which helps to reduce dye aggregation and decrease the recombination process between the injected electrons on the TiO_2_ semiconductor film and the electrolyte.^42,43^

### 3.3. Photovoltaic characterization of DSSCs

**Fig. 5:**
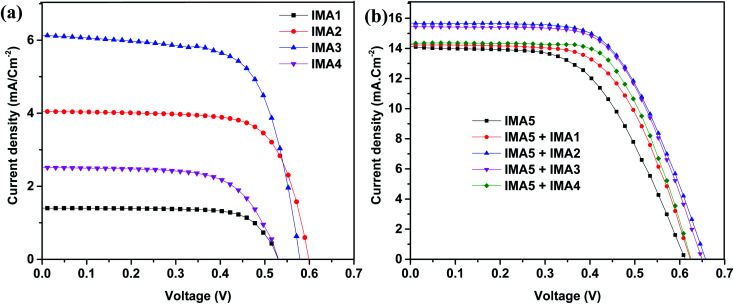
(a) *J*–*V* curves for the metal-free organic sensitizers. (b) *J*–*V* curves for the IMA5 complex alone and co-sensitized with IMA1-4 under 1.5 AM.

The DSSCs were fabricated utilizing dyes IMA1–5 with the addition of chenodeoxycholic acid (CDCA) as a co-adsorbent.^44^ The DSSCs were fabricated on FTO glass, which was printed with two layers of TiO_2_ and used as the working electrode (photoanode). The prepared photoanode was immersed into the dye/CDCA solution to allow the dye to anchor onto the surface. The counter electrode was prepared by printing Pt paste on FTO conductive glass. Both electrodes were sealed together and iodolyte (redox couple) solution was injected into the device interface; more details on the fabrication processes are provided in the ESI.† The photovoltaic properties of the fabricated DSSC devices sensitized with IMA5 and co-sensitizers IMA1–4 on a TiO_2_ semiconductor electrode were analyzed under standard AM 1.5 irradiation (100 mW cm^−2^). The resultant current–voltage (*J*–*V*) plots are presented in Fig. 5a and b and the related data are summarized in Table 2. From the results in Fig. 5a, IMA3 achieved the highest photocurrent efficiency of *η* = 2.41%, open circuit voltage (*V*_OC_ = 0.85 V), short-circuit current (*J*_SC_ = 6.13 mA cm^−2^) and fill factor (FF = 67.76%) when compared to photocurrent efficiencies (*η*) of 0.54%, 1.74%, and 0.88% for IMA1, IMA2, and IMA4, respectively. The photocurrent efficiencies of the organic dyes IMA1-4 were found to be in the order of IMA3 > IMA2 > IMA4 > IMA1, which is attributed to increasing electron injection from the excited dye molecules to the TiO_2_ surface of the DSSCs. The electron injection free energy (Δ*G*_inj_°) was found to have the same trend as photocurrent efficiency and was calculated from the difference between the CB of the TiO_2_ surface and ESOP, as shown in Table 1.

**Table 2:** Photovoltaic parameters of the sensitized/co-sensitized DSSCs for IMA1–5

Sensitizer (0.2 mM)	Co-sensitizer (0.2 mM)	No. of DSSCs	Average values (PV parameters)			
			*J*_SC_ (mA cm^−2^)	*V*_OC_ (V)	FF (%)	*η* (%)
IMA1	__	4	1.41	0.53	73.07	0.54
IMA2	__	4	4.05	0.59	71.49	1.74
IMA3	__	4	6.13	0.58	67.76	2.41
IMA4	__	4	2.51	0.53	65.79	0.88
IMA5	__	3	14.07	0.61	64.54	5.54
	IMA1	3	14.25	0.62	61.21	5.44
	IMA2	3	15.67	0.66	60.58	6.25
	IMA3	3	15.44	0.65	61.71	6.19
	IMA4	3	14.36	0.63	64.85	5.83

On the other hand, the relationship between the structures of the co-sensitizers IMA1–4 and their performance with ruthenium dye IMA5 was studied by evaluating the photovoltaic characterization of the co-sensitized DSSCs. The addition of different anchoring groups to the principal moiety of phenyl-DBT-phenyl was found to have a profound influence on the photovoltaic properties of the co-sensitized DSSC devices, as shown in Fig. 5b. The photovoltaic parameters of IMA5 alone were *η* = 5.54%, *V*_OC_ = 0.61 V, *J*_SC_ = 14.07 mA cm^−2^, whereas after employing co-sensitizers IMA1–4 the *J*_sc_ was enhanced to 14.25, 15.67, 15.44 and 14.36 mA cm^−2^ and the *V*_OC_ was enhanced to 0.62, 0.66, 0.65 and 0.63 V, respectively. The enhanced *J*_sc_ for IMA5 might be ascribed to the increased light harvesting in the 300–500 nm region owing to the addition of metal-free organic co-sensitizers IMA1-4, which have a characteristic high molar absorption coefficient. The improved *V*_OC_ observed for the co-sensitized DSSC devices can be attributed to the lower rate of recombination between the injected electrons in the TiO_2_ semiconductor conduction band and the redox electrolyte (*I*_3_^−^/*I*^−^). Owing to their small size, the co-sensitizers provide better surface coverage^27,45^ by adsorbing into the pores and gaps in the TiO_2_, whereas the steric hindrance of the bulky ruthenium-based dye molecule prevents its adsorption. From the results, the lower efficiency of IMA5 when co-sensitized with IMA1 (*η* = 5.44%) and IMA4 (*η* = 5.83%) compared to when co-sensitized with IMA2 (*η* = 6.25%) and IMA3 (*η* = 6.19%) can be attributed to the breaking of the continuous conjugation by the pyrazole moiety in IMA1 and by the thiazole moiety in the case of IMA4, which would prevent efficient electron injection into the conduction band of TiO_2_.^46^ Among all the DSSCs, the DSSC device co-sensitized with IMA2 showed the highest *J*_sc_, *V*_OC_, and PCE owing to the small size of IMA2, which includes a cyanoacetic acid moiety. These criteria increase the adsorption of the molecule on the TiO_2_ surface and enhance its light-harvesting ability.

**Fig. 6:**
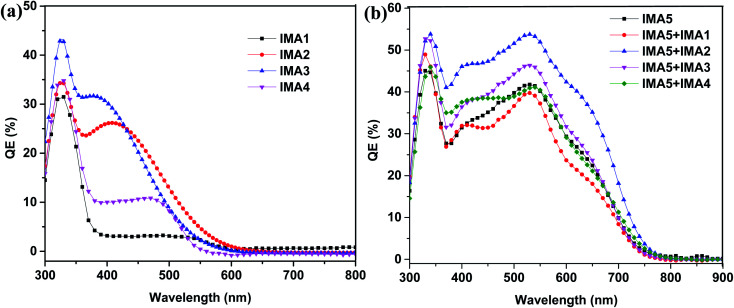
(a) IPCE curves for IMA1–4. (b) IPCE curves for IMA5 complex alone and co-sensitized with IMA1–4.

The IPCEs for the DSSCs sensitized with IMA1–5 and the DSSCs sensitized with IMA5 and co-sensitized with IMA1–4 were measured and are plotted in Fig. 6a and b. The DSSCs sensitized with the metal-free organic dyes IMA1–4 showed IPCE spectra in the visible range (300–500 nm) with quantum efficiencies of 4%, 27%, 33% and 12%, respectively, as shown in Fig. 6a. The higher IPCE of IMA3 compared to the other dyes is attributed to the high molar extinction coefficient of IMA3 (0.953 × 10^5^ M^−1^ cm^−1^). On the other hand, the DSSC devices with IMA5 alone and co-sensitized with IMA1–4 show broad coverage of IPCE spectra from the visible range to the near IR range (300–700 nm) with quantum efficiencies of 40%, 54%, 47%, and 41%, respectively, compared to 42% for IMA5 alone, as shown in Fig. 6b. The improved IPCEs of the DSSCs co-sensitized with IMA2 and IMA3 compared to IMA5 alone is attributed to the increased electron injection ability with co-sensitizers incorporating cyanoacetic acid and quinoline carboxylic acid moieties. While, the lower IPCEs of the DSSCs co-sensitized with IMA1 and IMA4 is because of the low electron injection efficiency owing to the disrupting of the conjugation by the pyrazole and thiazole moieties and the unfavorable LUMO distribution.^46^

### 3.4. Electrochemical impedance study of the DSSCs

**Fig. 7:**
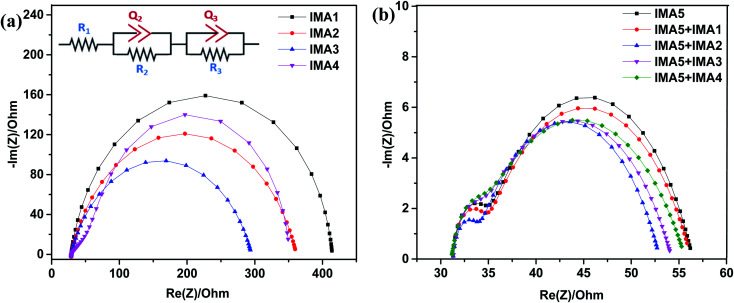
(a) Nyquist plots of DSSCs sensitized with IMA1–4 and equivalent circuit for *R*_1_+ *C*_2_/*R*_2_ + *C*_3_/*R*_3_ fitting model. (b) Nyquist plots of DSSCs sensitized with IMA5 and co-sensitized with IMA1–4.

Electrochemical impedance spectroscopy was conducted to explain the recombination rate and charge lifetime of the DSSCs sensitized/co-sensitized with sensitizers IMA1–5. The Nyquist and Bode plots for the devices with IMA1–4 are shown in Fig. 7a and b and the corresponding data are summarized in Table 3. The equivalent circuit fitting was performed using (Bio-Logic) software utilizing the *R*_1_+ *C*_2_/*R*_2_ + *C*_3_/*R*_3_ fitting model as shown in Fig. 7a. In the EIS Nyquist plots, three semicircles were observed, the first and small semicircle in the higher frequency region corresponds to the charge transfer process (*R*_3_) at the counter electrode and electrolyte interfaces. The large or mid-frequency region signifies the recombination resistance (*R*_2_) at the working electrode of the TiO_2_/dye/electrolyte interface.^27,46^ The third semicircle (low-frequency range) indicates the series resistance (*R*_1_) of platinum and FTO glass. The obtained recombination resistance values (*R*_2_) of the DSSCs sensitized with IMA1–4 were 8.605, 64.75, 77.37, and 40.33 Ω, respectively. In fact, the higher the *R*_2_ value, the lower the charge recombination rate between the conduction band of the TiO_2_ semiconductor and the electrolyte. The *R*_2_ values of the DSSC devices sensitized with IMA1–4 decreased in the order of IMA3 > IMA2 > IMA4 > IMA1. The obtained *R*_2_ values were consistent with the *V*_OC_ values obtained from the *J*–*V* curves. Among all of the sensitizers, IMA3 achieved the highest *R*_2_ value which enhancing the charge recombination resistance between the semiconductor conduction band and the electrolyte compared to the other sensitizers, which indicates that quinoline carboxylic acid is an efficient anchoring group. On the other hand, the EIS Nyquist plots of the DSSCs sensitized with IMA5 alone and co-sensitized with IMA1–4 showed recombination resistance (*R*_2_) values in the order of IMA5+IMA2 > IMA5+IMA3 > IMA5+IMA4 > IMA5+IMA1 > IMA5. The *R*_2_ values followed the same trend as the *V*_OC_ values obtained from the *J*–*V* curves. All co-sensitizers achieved higher recombination resistance (*R*_2_) compared to IMA5 alone. Among all the co-sensitizers, IMA2 achieved the highest *R*_2_ value which enhancing the charge recombination resistance compared to other co-sensitizers, which indicates that dyes incorporating cyanoacetic acid anchoring groups are efficient co-sensitizers.

**Table 3:** Parameters obtained from applying the *R*_1_+ *C*_2_/*R*_2_ + *C*_3_/*R*_3_ fitting model on the impedance spectra of the sensitized/co-sensitized DSSCs^a^

Parameters	Sensitized DSSC devices					Co-sensitized DSSC devices			
	IMA1	IMA2	IMA3	IMA4	IMA5	IMA5+IMA1	IMA5+IMA2	IMA5+IMA3	IMA5+IMA4
*R*_1_ (Ω cm^−2^)	24.96	41.22	23.15	28.81	30.85	26.59	25	28.56	26.88
*C*_2_ (μF cm^−2^)	357	323	159	851	258	357	232	140	157
*n*_1_	1	0.993	1	0.553	0.672	0.628	0.518	0.637	0.577
*R*_2_ (Ω cm^−2^)	8.605	64.75	77.37	40.33	21.93	22.13	22.65	22.52	22.48
*C*_3_ (μF cm^−2^)	25	28	33	286	11.57	10.67	29.84	12.65	17.15
*n*_2_	0.877	0.807	0.785	0.966	0.974	1	1	0.942	1
*R*_3_ (Ω cm^−2^)	379.4	264.7	265.4	283.7	3.25	2.92	7.65	2.95	2.17
*τ*_eff_ (ms)	1.29	1.56	2.45	1.39	2.85	2.96	6.43	4.50	3.33

a *R*_1_, *R*_2_, and *R*_3_ are the series resistance of Pt and TCO, the charge transfer resistance at the TiO_2_/dye/electrolyte interface, and the charge transfer resistance at the Pt/electrolyte interface, respectively; *C*_2_ and *C*_3_ are the constant phase elements for the TiO_2_/dye/electrolyte and Pt/electrolyte interface, respectively; *n* presents the degree of surface inhomogeneity; *τ*_eff_ is the effective lifetime.

**Fig. 8:**
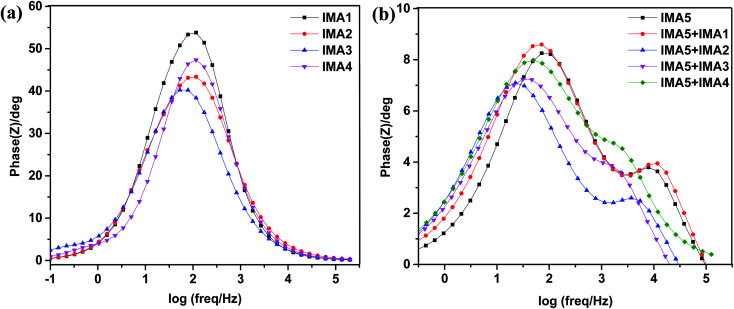
(a) Bode plots of DSSCs sensitized with IMA1–4. (b) Bode plots of DSSCs sensitized with IMA5 and co-sensitized with IMA1–4.

Furthermore, the Bode phase plots were analyzed to determine the electron recombination lifetime in the CB of TiO_2_, as shown in Fig. 8a and b. The frequency peaks (*f*) obtained from the DSSCs sensitized with IMA1–4 in the Bode phase plots were used to calculate the effective lifetime (^τ^*τ*_eff_) of electrons injected into the CB of TiO_2_ by utilizing the following relation: 
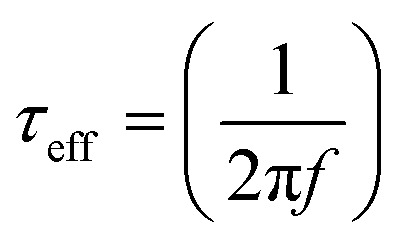
. The effective lifetimes, *τ*_eff_, for the devices sensitized with IMA1–4 were calculated and found to decrease in the following order: IMA3 (2.45 ms) > IMA2 (1.56 ms) > IMA4 (1.39 ms) > IMA1 (1.29 ms). The effective lifetimes, *τ*_eff_, for the DSSCs sensitized with IMA5 alone and co-sensitized with IMA1–4 showed the following order: IMA5+IMA2 > IMA5+IMA3 > IMA5+IMA4 > IMA5+IMA1 > IMA5. Basically the longer electron lifetime leads to a better suppression of back reactions between the injected electrons and the electrolyte, which usually leads to improvement of the *V*_OC_.^47–49^

The *V*_OC_ values of the co-sensitized devices obviously follow the same order as the *τ*_eff_ values, clearly showing the great effect of anchoring groups and co-sensitization on the electron recombination processes between the electrolyte species and electron transfer into the TiO_2_ semiconductor film. Hence, the decrease in the *V*_OC_ for the IMA5 device can be explained by the faster recombination relative to that of the IMA5+IMA2 co-sensitized device, which can be attributed to the increase in dye loading on the surface of the TiO_2_ by the adsorption of the larger IMA5 complex followed by the adsorption of smaller IMA1–4 molecules in such a way as to fill the gaps between larger IMA5 molecules in the sensitization process, which helps with the formation of a blocking layer covering the complete TiO_2_ nanoparticle owing to increased dye loading.^47,50^

## 4. Conclusions

In summary, a new series of organic sensitizers IMA1–4 with A–π–D–π–A motif were synthesized and discussed along with the Ru(ii) complex IMA5 to be used as effective sensitizers/co-sensitizers for DSSCs and their performance, photophysics, electrochemistry and molecular modeling were compared. The DSSCs fabricated with sensitizers IMA1–4 showed PCE performance that decreased in the order of IMA3 > IMA2 > IMA4 > IMA1, which depicts the anchoring nature (2-methylquinoline-6-carboxylic acid, cyanoacetic acid, rhodamine-3-acetic acid, and 1-phenyl-pyrazol-5-one-3-carboxylic acid). Among these sensitizers, IMA3 showed the highest PCE, which can be ascribed to the enhanced light harvesting coupled with better electron injection into the TiO_2_ conduction band, and, hence, high electron injection efficiency owing to the presence of the quinoline ring between the anchoring group and the DBT center moiety. On the other hand, the short-circuit electron density (*J*_SC_) of the DSSCs fabricated with the IMA5 complex was significantly enhanced from 14.25 mA cm^−2^ to 14.25, 15.67, 15.44 and 14.36 mA cm^−2^, respectively when IMA1–4 were used as co-sensitizers. The corresponding enhancement in the *J*_SC_ could be attributed to the increased IPCE in the 300–600 nm range, possibly owing to the complementary absorption properties of the co-sensitizers (IMA1–4) with the IMA5 complex, thereby harvesting a larger number of photons in this region, which in turn enhanced the *J*_SC_ and PCE. Moreover, improved *V*_OC_ values were observed for the IMA5 DSSC devices when co-sensitized with IMA1–4 compared to the DSSC using the IMA5 dye alone, most likely owing to the small size of the co-sensitizers, which may occupy pores and gaps between the 3D ruthenium-based dye, which plays a key role in suppressing charge recombination by acting as a physical insulator between the TiO_2_ semiconductor and the *I*_3_^−^ electrolyte ions, which was confirmed by the EIS studies.

## Conflicts of interest

There are no conflicts of interest to declare.

## Supplementary Material

RA-010-D0RA03916K-s001
