# Supplementary material for: Efficiency enhancement of ruthenium-based DSSCs employing A–π–D–π–A organic Co-sensitizers
Source: RSC Adv. 2020 Jul 27;10(47):27940–53. doi: 10.1039/d0ra03916k (PMC9127656; doi:10.1039/d0ra03916k)
Supplement: RA-010-D0RA03916K-s001 [file RA-010-D0RA03916K-s001.pdf]

Electronic Supplementary Material (ESI) for RSC Advances.
This journal is © The Royal Society of Chemistry 2020

Supplementary Information

Efficiency Enhancement of Ruthenium Based DSSCs Employing A-π-D-π-A Organic Co-sensitizers

Islam M. Abdellah[1] and Ahmed El-Shafei[2*]

[1]*Department of Chemistry, Faculty of Science, Aswan University, Aswan, Egypt 81528*

[2]*Polymer and Color Chemistry Program, North Carolina State University, Raleigh, USA 27606*

***Corresponding Author**: Ahmed_El-Shafei@ncsu.edu.

1. Device fabrication

1.1. Device fabrication for IMA1-4 dyes

Fluorine-doped tin oxide (FTO) coated glass (2.2 mm thickness, sheet resistance of 8 Ω/cm^2, TEC, Pilkington) were washed with detergent, water, acetone and ethanol, respectively. Then, FTO glass plates are immersed in a 40 mM aqueous $TiCl_4$ solution at 70 °C for 30 minutes and washed with water and ethanol. A thin layer (8-12 μm thick) of TiO_2 (Solaronix, Ti-Nanoxide D/SP) (active area, 0.18 cm^2) was deposited on transparent conducting glass by printing, then dried at 350 °C for 10 minutes and curing at 500 °C for 30 min. Then, after the electrodes are dried, the TiO_2 particles of the scattering layer (5 μm thick) (Solaronix, Ti-Nanoxide R/SP) are printed on the already deposited TiO2 layer. TiO_2 electrodes were heated under airflow at 350 ° C for 10 minutes, then heated at 500 ° C for 30 minutes. After cooling to room temperature, the TiO_2 electrodes were treated with 40 mM aqueous solution of $TiCl_4$ at 70 °C for 30 minute and then washed off with water and ethanol. The electrodes were heated again at 500 ° C for 30 minutes, and allowed to cool to 80 ° C before dipping in IMA1-4 solutions. The IMA1-4 solutions (0.2 mM) were prepared in 10 mL 1: 1 acetonitrile and tert-butanol. Chenodeoxycholic acid (CDCA) was added at a concentration of 20 mM. The electrodes were immersed in IMA5 solution (0.2 mM) and then kept

at 25 ° C for 20 hours to adsorb IMA1-4 on the TiO_2 surface. The counter electrode was prepared from pre-cut TCO glass (transparent conductive oxides) washed with water followed by 0.1M HCl in EtOH, and then sonication in an acetone bath for 10 min. These washed TCO was then dried at 400 °C for 15 minutes. A thin layer of Pt paste (Solaronix, Platisol T/SP) was printed on TCO and the printed electrodes were cured at 450 ° C for 10 minutes. The dye sensitized TiO_2 electrodes were sandwiched with Pt counter electrodes and the electrolyte (Solaronix, Iodolyte HI-30) was injected into the cells, while the two electrodes were held together with clips.

1.2. Device fabrication for IMA5 complex

The fabrication was performed using the same procedure as IMA1-4 devices fabrication utilizing a thin layer (8-12 μm thick) of TiO_2 (Solaronix, Ti-Nanoxide T/SP) was deposited instead of TiO_2 (Solaronix, Ti-Nanoxide D/SP).

1.3. Device fabrication for the co-sensitized devices

The fabrication was performed using the same procedure as IMA1-4 devices fabrication. While the TiO_2 electrodes were immersed in a mixture of IMA5 (0.2 mM) and IMA1-4 (0.2 mM).

2. Synthesis

2.1. Synthesis of 2, 2’-Bipyridinyl-4, 4’-dicarboxylic acid

The 2, 2’-Bipyridinyl-4, 4’-dicarboxylic acid was synthesized according to published procedure [3]. In a three neck round bottom flask 4,4’-Dimethyl-2,2’-bipyridine (3 g, 16.3 mmol) was gradually added to a stirred solution of sodium dichromate ($Na_2Cr_2O_7$) (10.9g, 36.5 mmol) in concentrated sulfuric acid (H_2SO_4) (93%, 45 ml) and stirred for 30 min at 75°C. After 30 min, the dark green reaction mixture was poured into cold water forming a light-yellow precipitate. The precipitate was then filtered under vacuum and dissolved in 10 % aq. Sodium hydroxide. The product was crystallized by slowly acidifying the solution with 10 % aq. hydrochloric acid at a pH

of 2. The precipitation and acidification process were repeated three times to obtain the white chromium free precipitates of 2, 2'-Bipyridinyl-4, 4'-dicarboxylic acid (2.56g, 90%).

2.2. N,N-diphenyl-4-(4,4,5,5-tetramethyl-1,3,2-dioxaborolan-2-yl)aniline (7)

To a three-necked flask adding 1,4-dioxane solution of 4-bromo triphenyl amine (5 g, 15.42 mmol), bis(pinacolato)diboron (4.7 g, 18.5 mmol) and CH_3COOK (4.54 g, 46.62 mmol), The solution was purged with argon for 30 min, and then Pd(dppf)Cl_2 (0.38 g, 0.465 mmol) was added. The reaction was stirred at 85°C overnight. The reaction followed by TLC until reaction completion, then let the reaction to cool down and the reaction quenched by adding (50ml) of water then extracted by ethyl acetate (3 x 30 ml) The combined organic layers were dried over anhydrous Mg_2SO_4, and the solvent was removed under vacuum. The crude product was purified by column chromatography on silica with hexane/ethyl acetate mixture (6: 1). The compound crystalized from anhydrous ethanol and give the pure white powder. Yield (80 %), melting point (m.p) 93 °C.

2.3. Synthesis of 2-(3,4-Ethylenedioxythiophene) carbaldehyde

3,4-Ethylenedioxythiophene (25 g, 0.1755 mmol) was dissolved in dry DMF (100 mL). The mixture was cooled to -10 °C and $POCl_3$ (17 mL, 0.18 mmol) was added drop-wise (15 min) in the cold solution. The mixture stirred 1 h at -10 °C, ice water (200 mL) was added and the mixture stirred overnight at room temperature. The aldehyde was filtered off, dissolved in CH_2Cl_2 (200 mL) and dried (Na_2SO_4). The CH_2Cl_2 filtrate was eluted through a short silica plug to remove colored byproducts, giving the dry product as white crystals (21g, 71%).

2.4. Synthesis of 2-Bromo-(3,4-ethylenedioxythiophene)-5-carbaldehyde (8).

2-(3,4-Ethylenedioxythiophene) carbaldehyde (4.04 g, 23.7 mmol) was suspended in dry acetonitrile (100 mL) and cooled to 0 °C. NBS (4.36 g, 26.0 mmol), 1.1 equiv, was added and the mixture was stirred for 60 hrs at room temperature, shielded from light and under nitrogen. The

color changed from yellow to purple. The mixture was transferred with 150 mL of ethyl acetate to a separation funnel, washed with 10% aqueous Na_2CO_3 (2×200 mL), saturated $Na_2S_2O_3$ (2×200 mL) and water (2×200 mL), dried with $MgSO_4$, and evaporated in vacuo. Recrystallization twice from ethanol (60 mL) of the bromide as yellow needles (5.35 g, 91%).

3. Electrochemical measurements (CV)

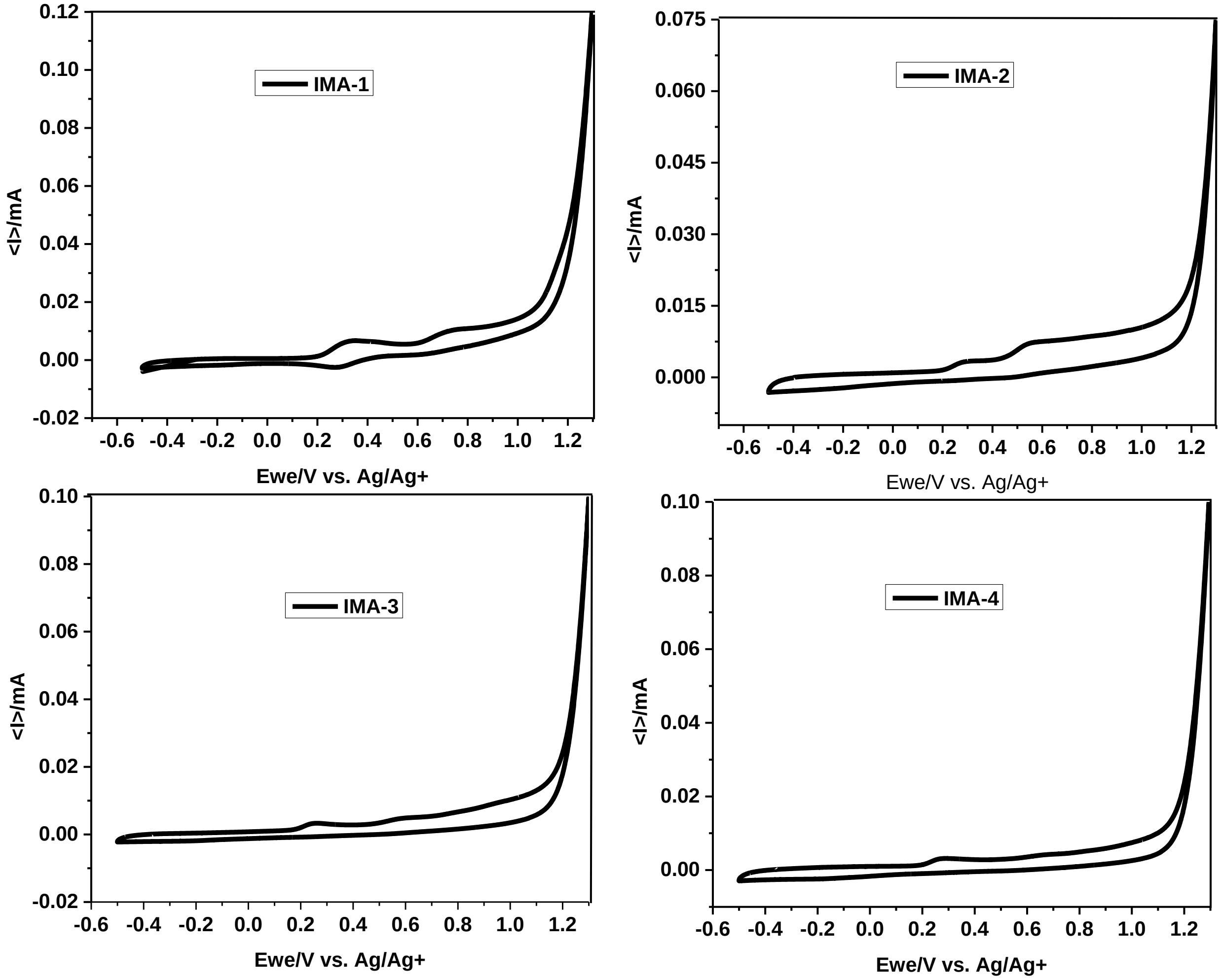

Fig. S1. Cyclic voltammogram of **IMA-(1-4)** in DMSO solution

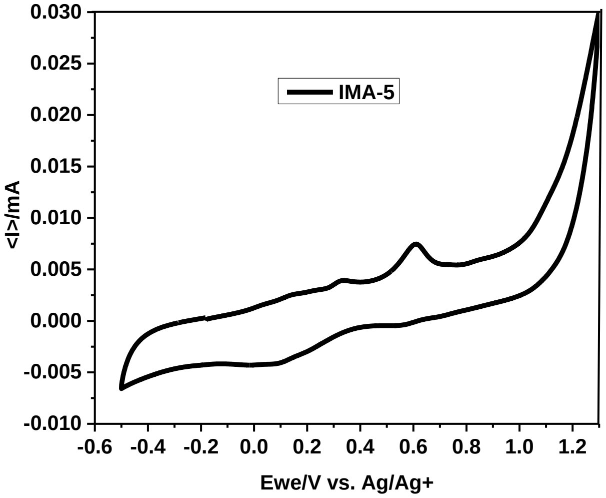

Fig. S2. Cyclic voltammogram of **IMA-5** complex in chloroform solution

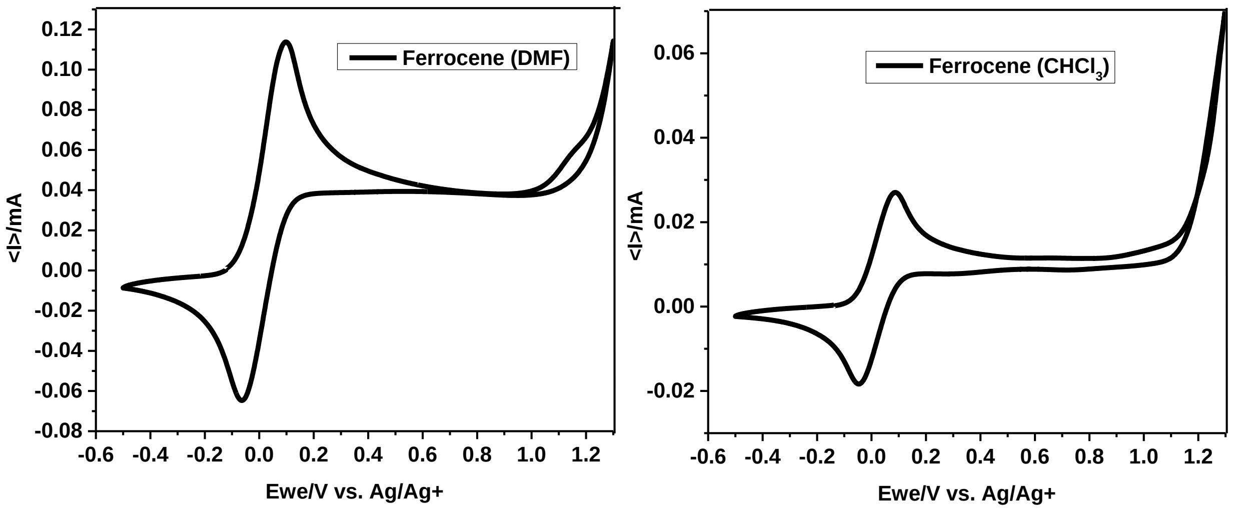

Fig. S3. Cyclic voltammogram of ferrocene compound in DMF/$CHCl_3$ solution

4. Spectral results

4.1.High resolution mass spectra

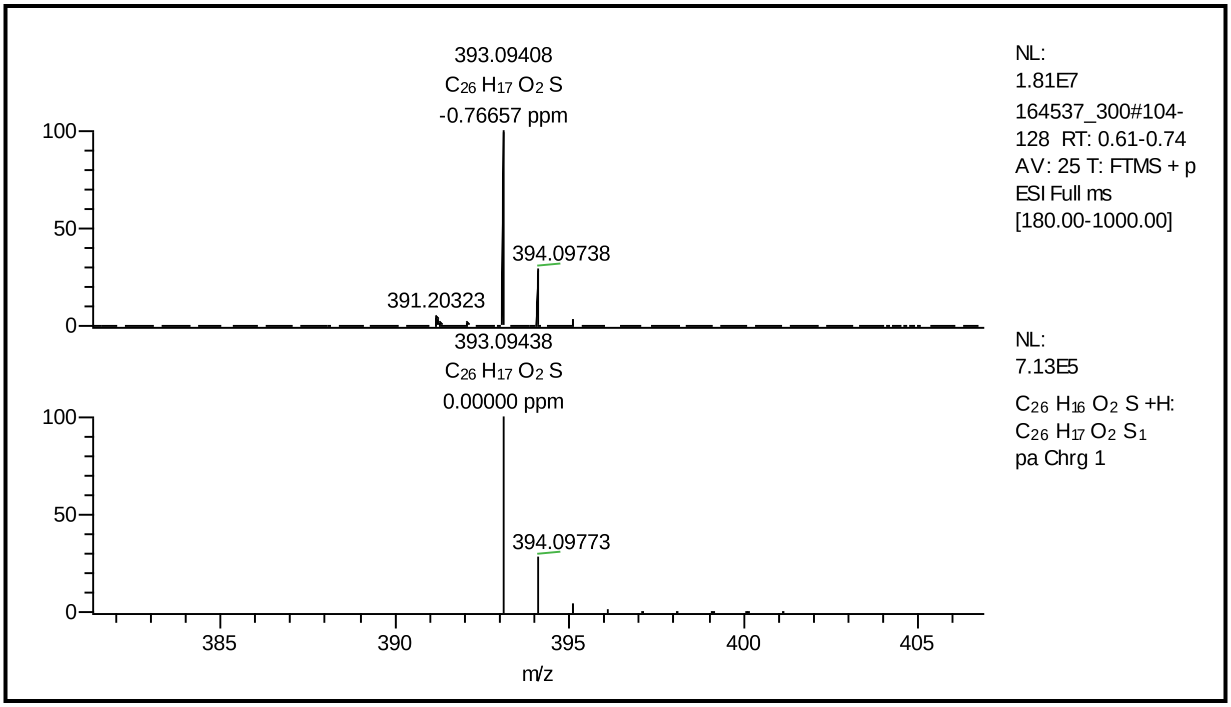

Fig. S4. Experimental and Theoretical Isotopic Distribution for **2**, $[M+H]^+$

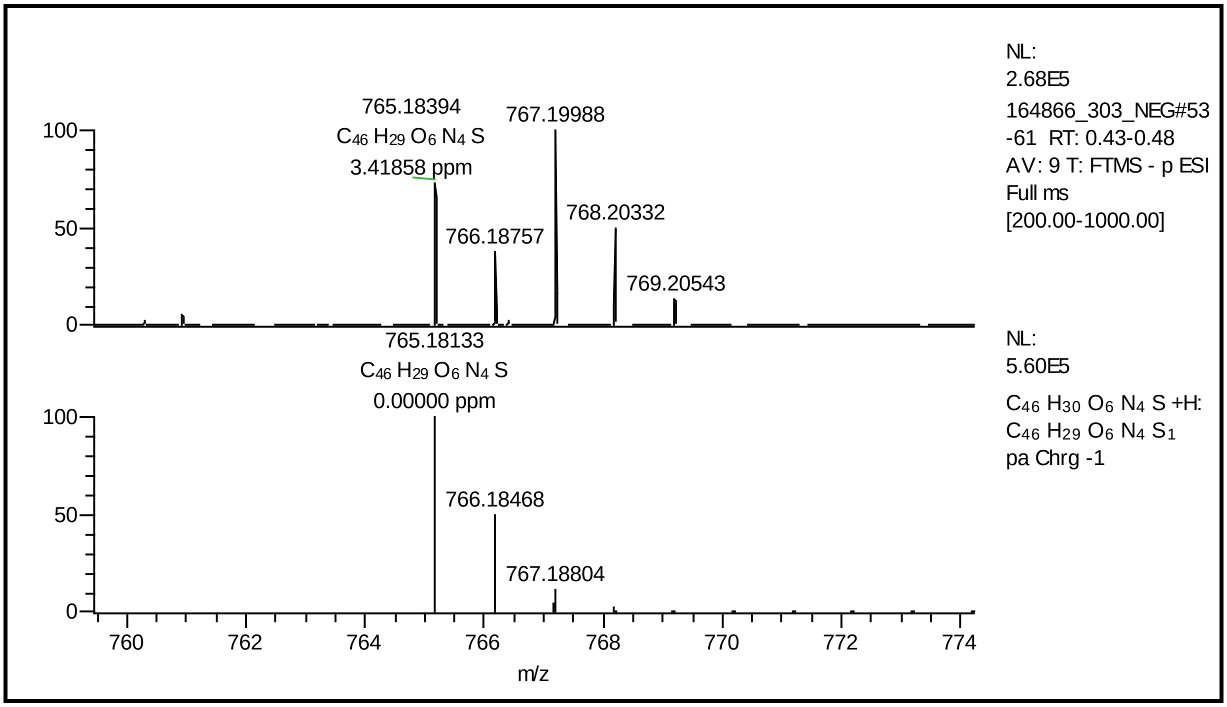

Fig. S5. Experimental and Theoretical Isotopic Distribution for **IMA1**, $[M-H]^-$

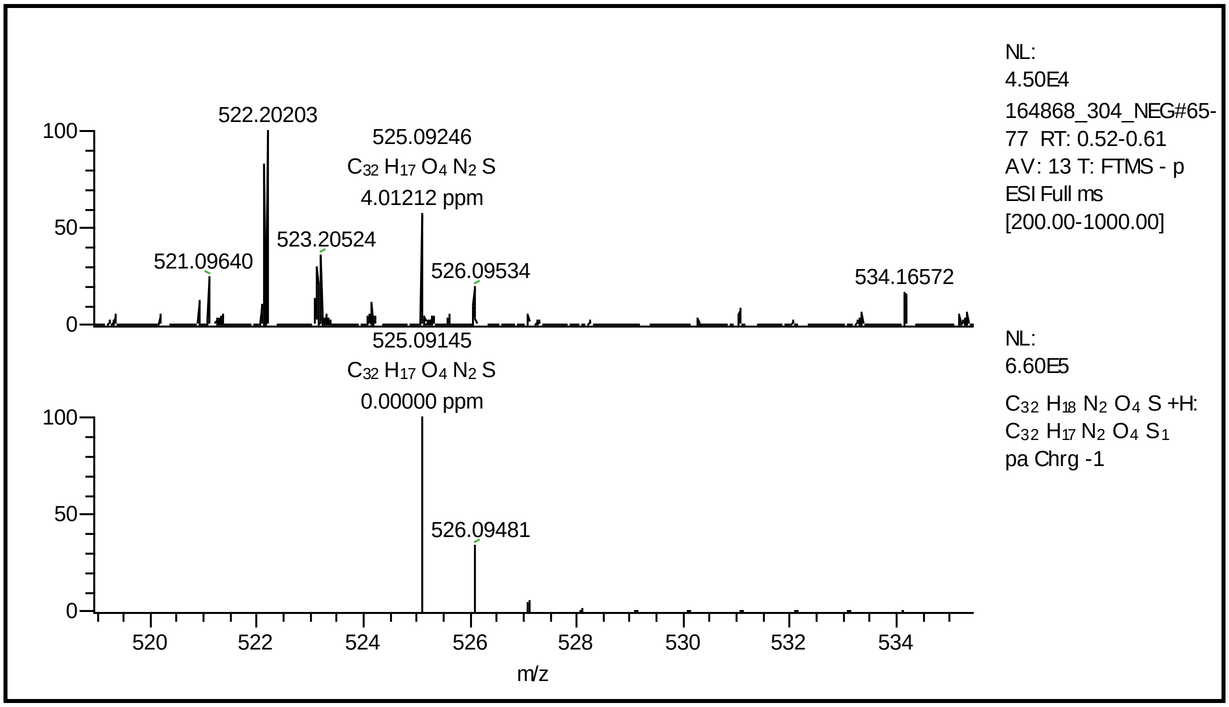

Fig. S6. Experimental and Theoretical Isotopic Distribution for **IMA2**, $[M-H]^-$

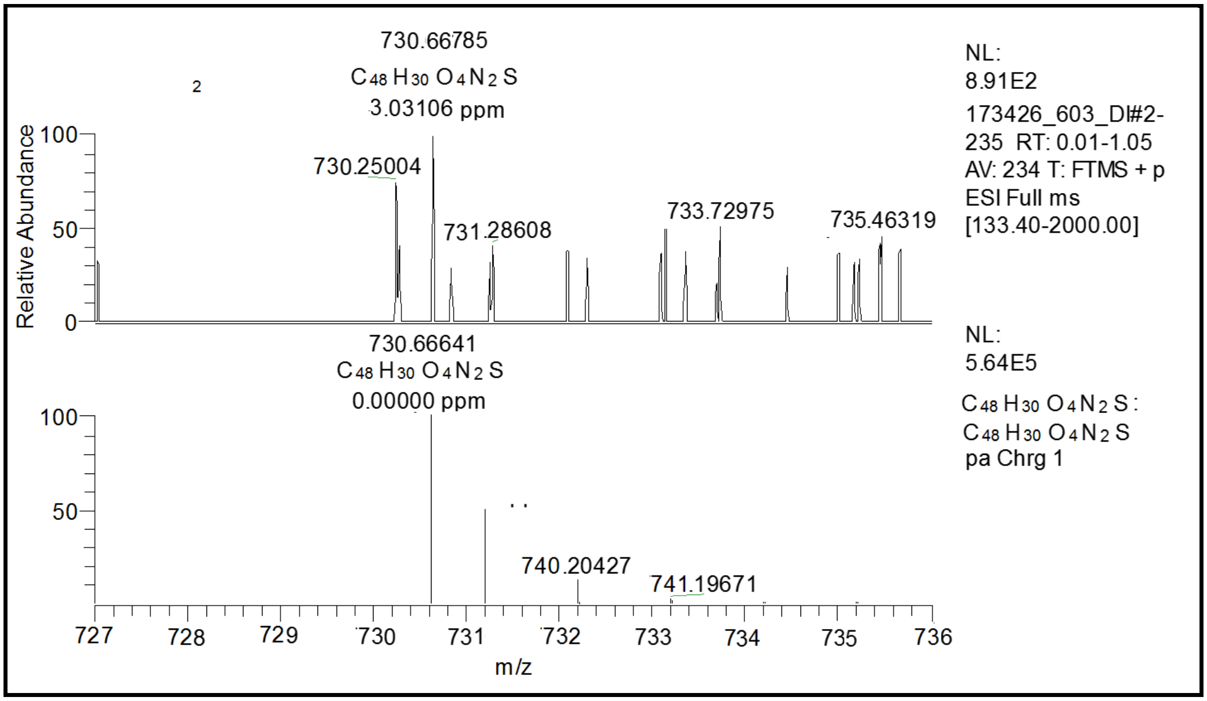

Fig. S7. Experimental and Theoretical Isotopic Distribution for **IMA-3**, $[M]^+$

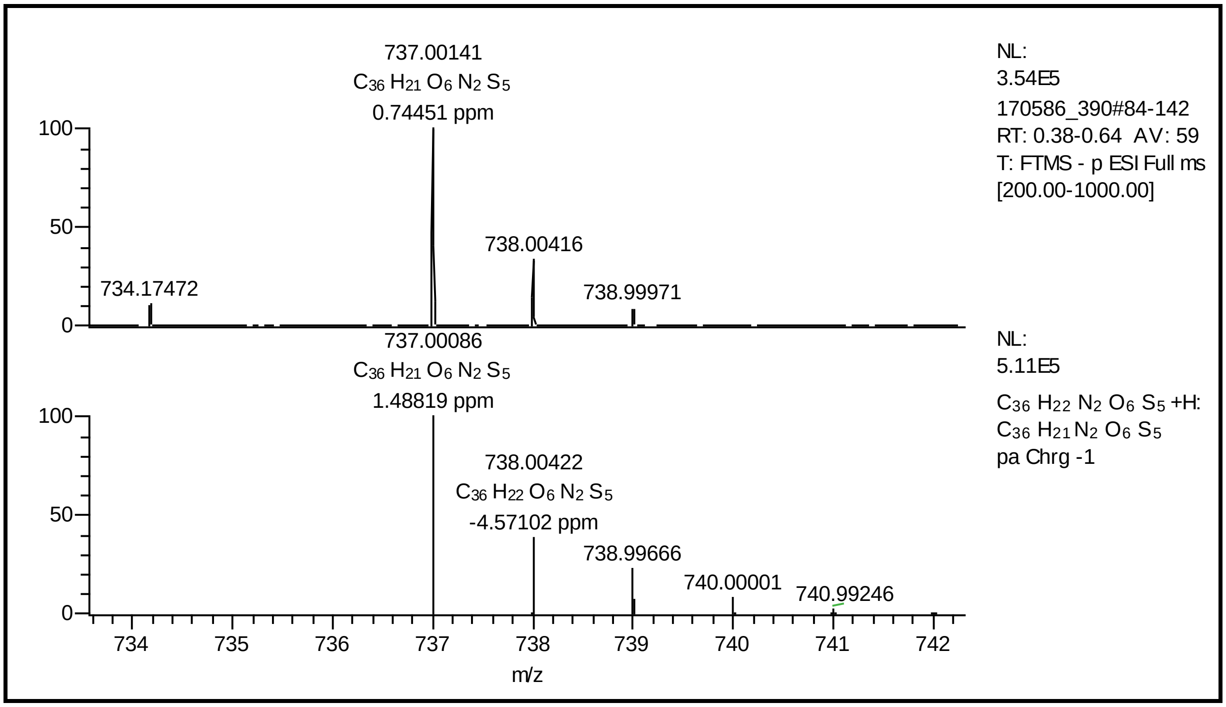

Fig. S8. Experimental and Theoretical Isotopic Distribution for **IMA4**, $[M-H]^-$

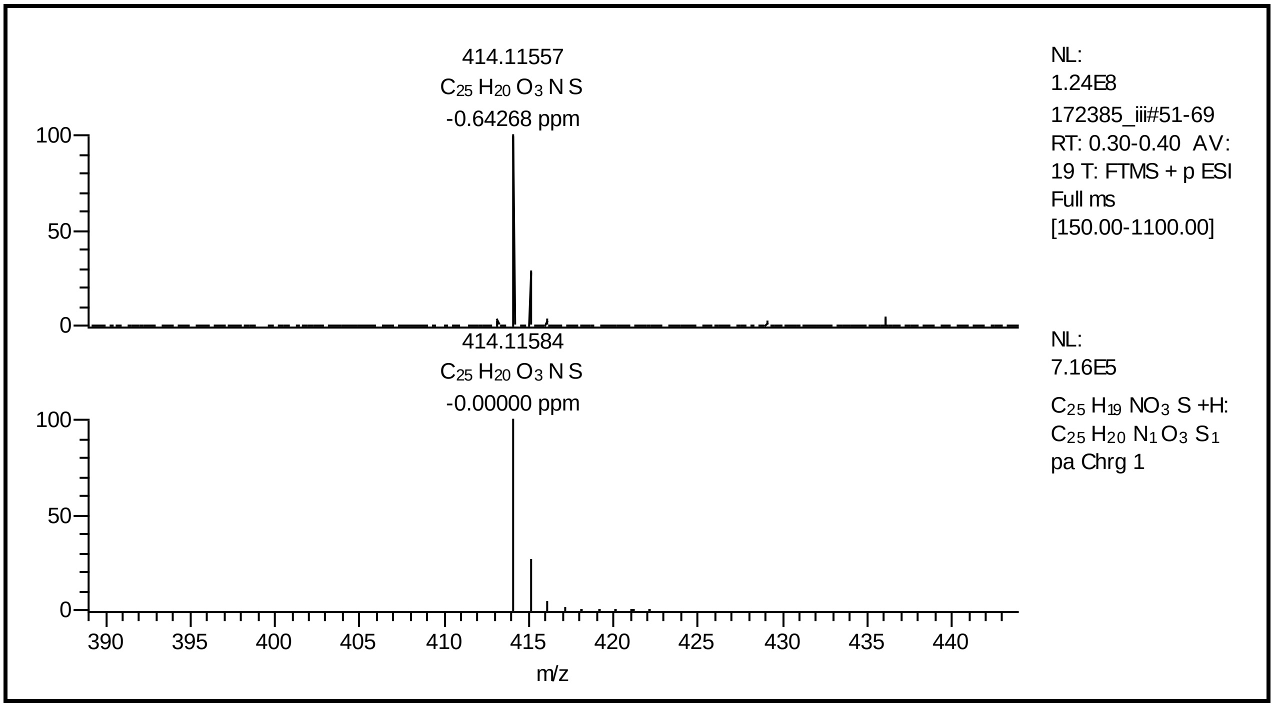

Fig. S9. Experimental and Theoretical Isotopic Distribution for **9**, **[M+H]$^+$**

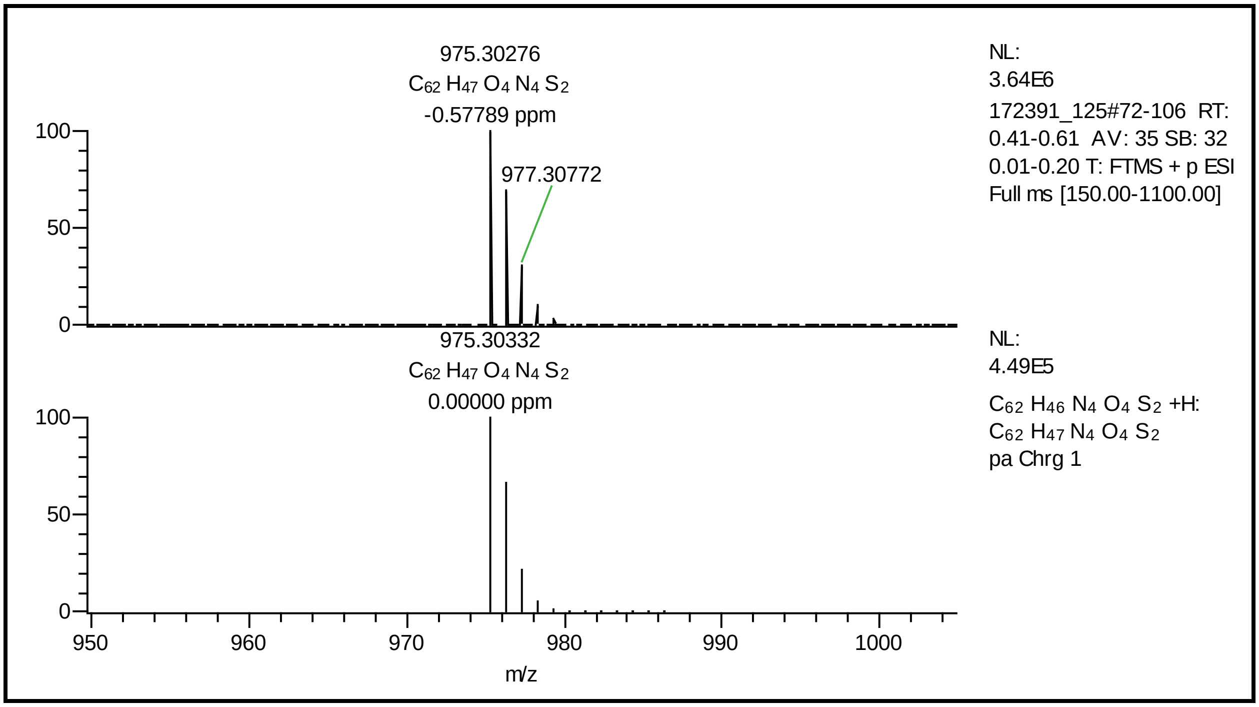

Fig. S10. Experimental and Theoretical Isotopic Distribution for **10**, **[M+H]$^+$**

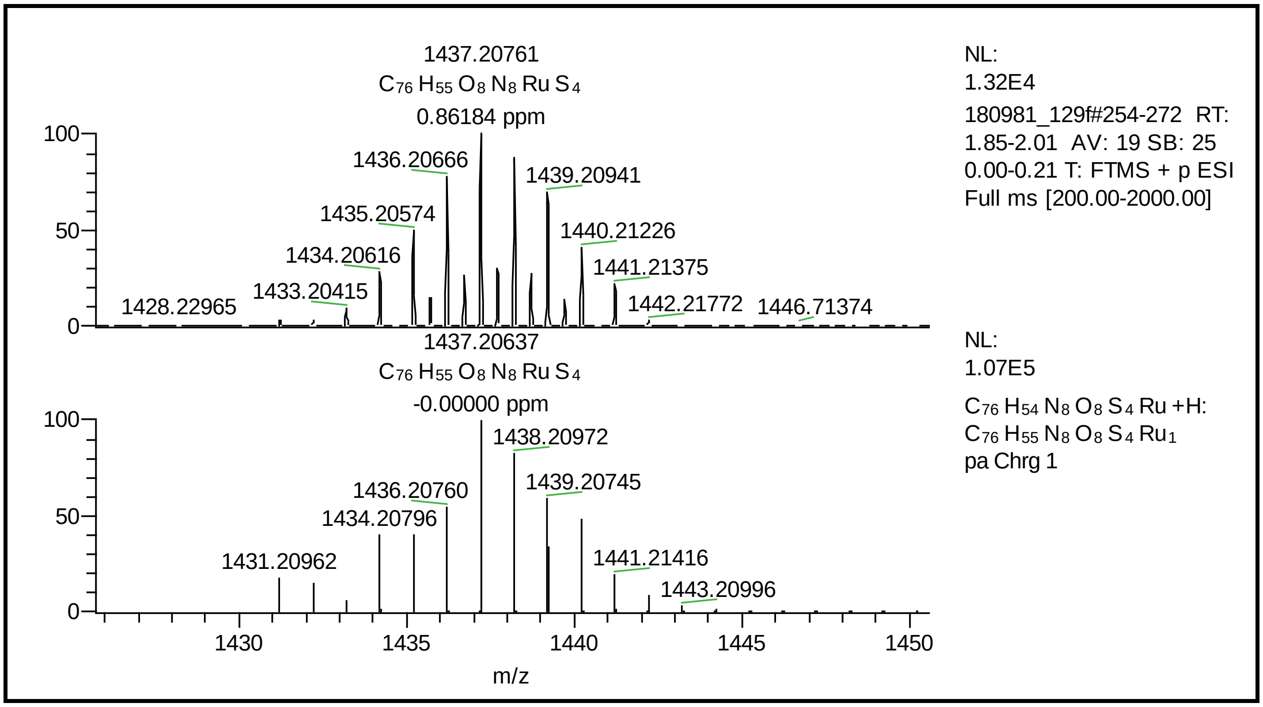

Fig. S11. Experimental and Theoretical Isotopic Distribution **IMA5**, $[M+H]^+$

4.2. FTIR Spectra

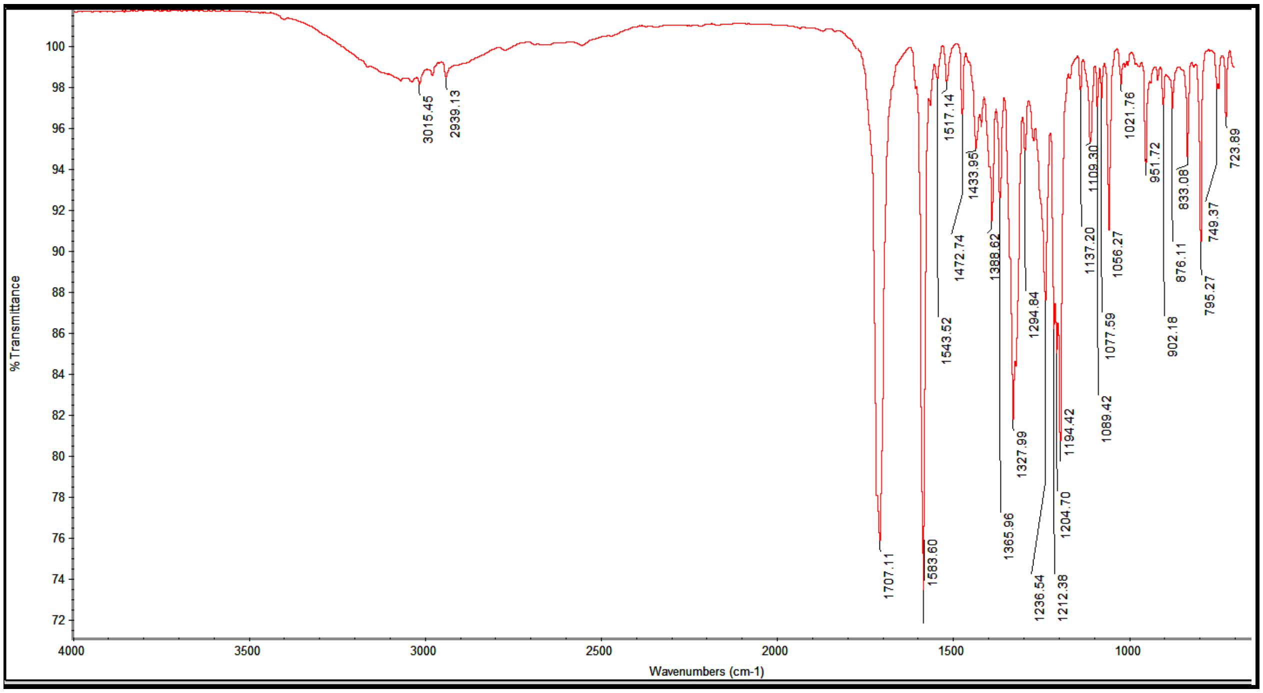

Fig. S12. FTIR spectra for **2**

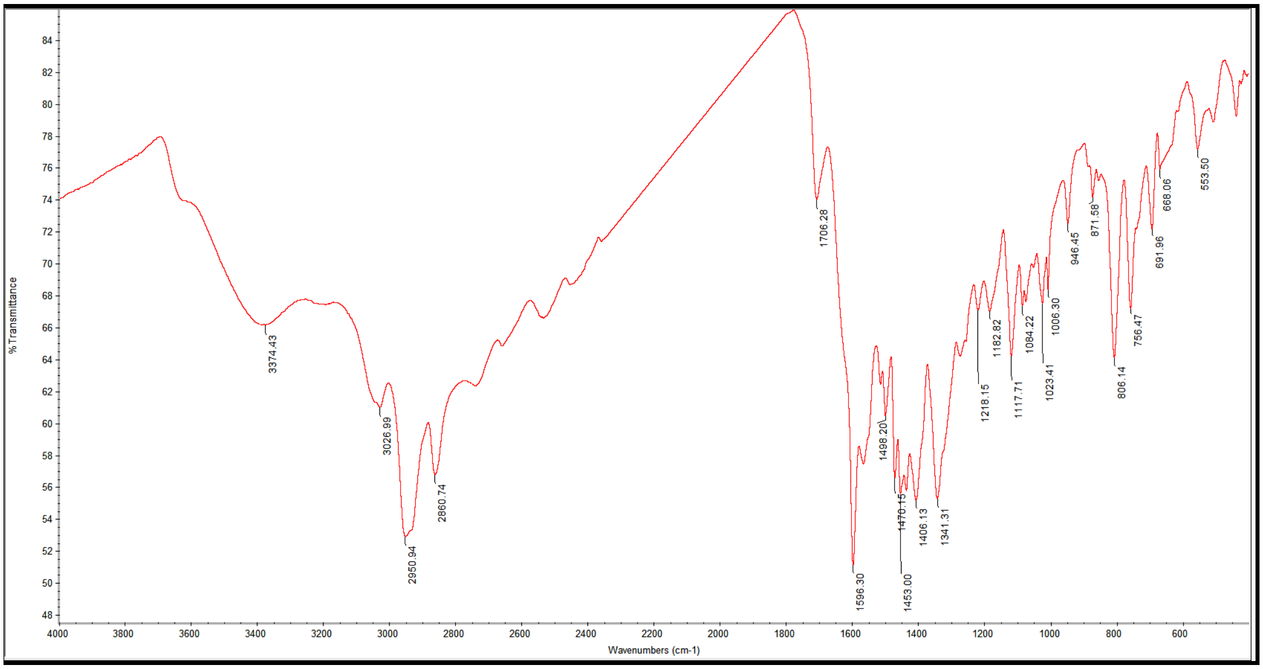

Fig. S13. FTIR spectra for **IMA1**

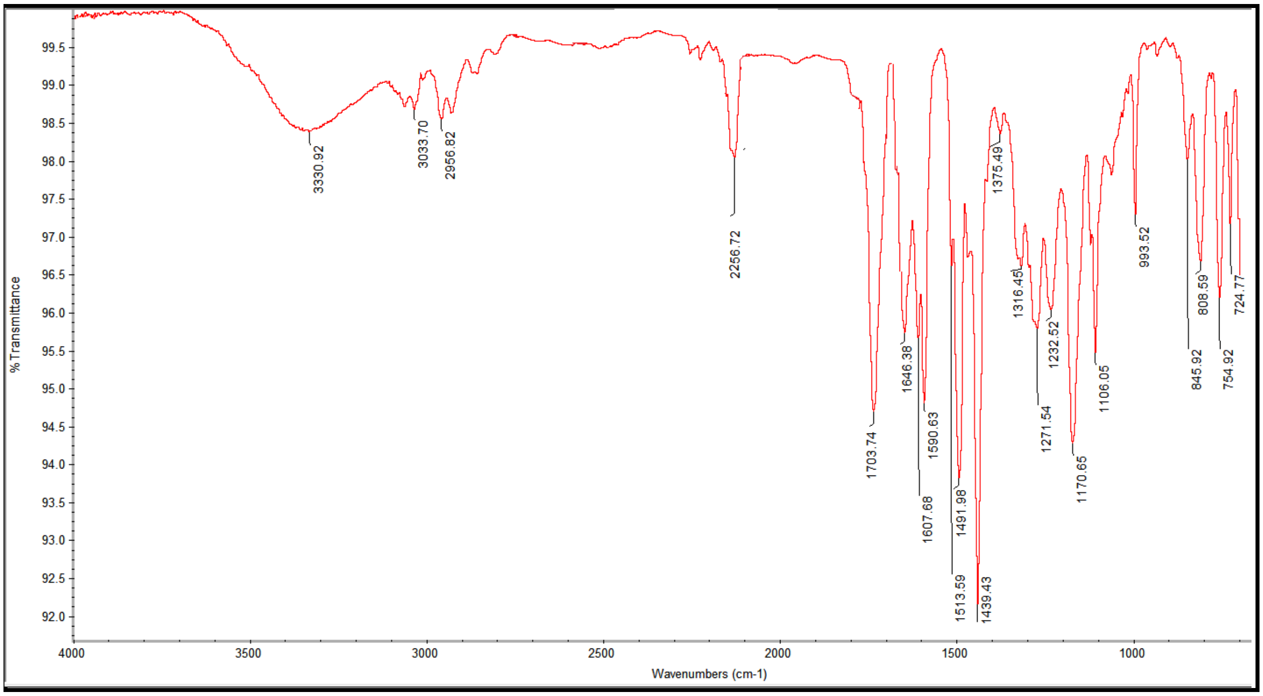

Fig. S14. FTIR spectra for **IMA2**

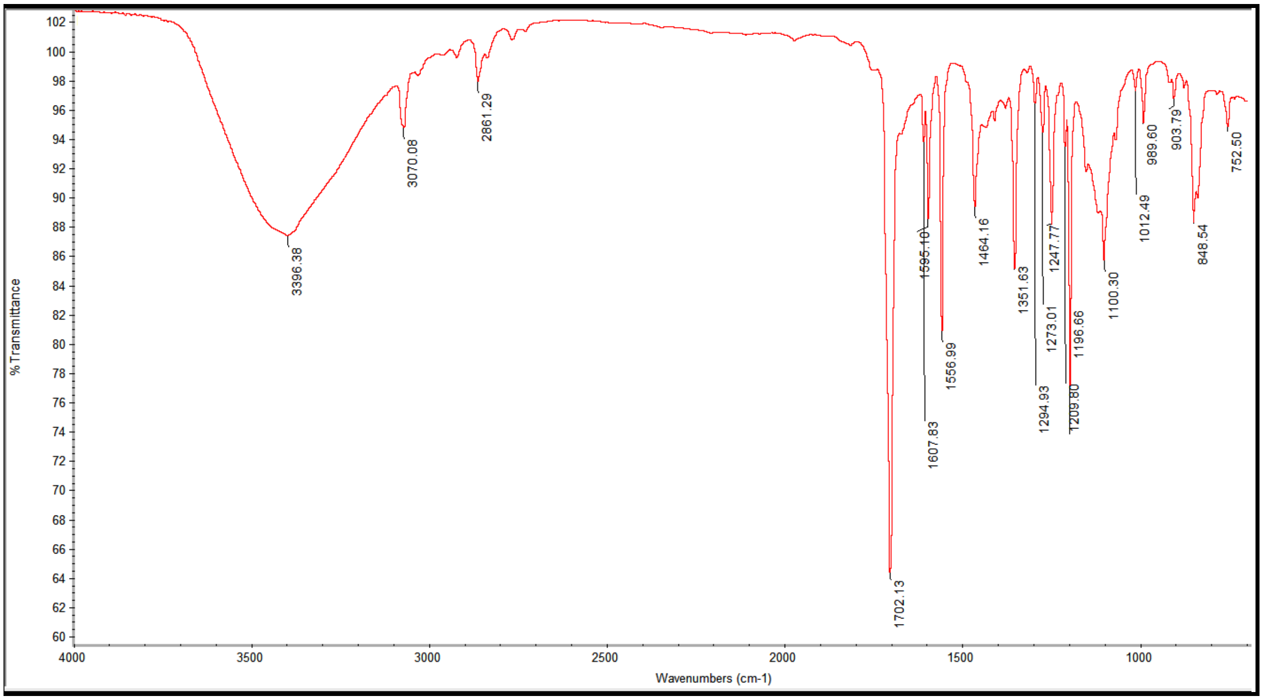

Fig. S15. FTIR spectra for **IMA3**

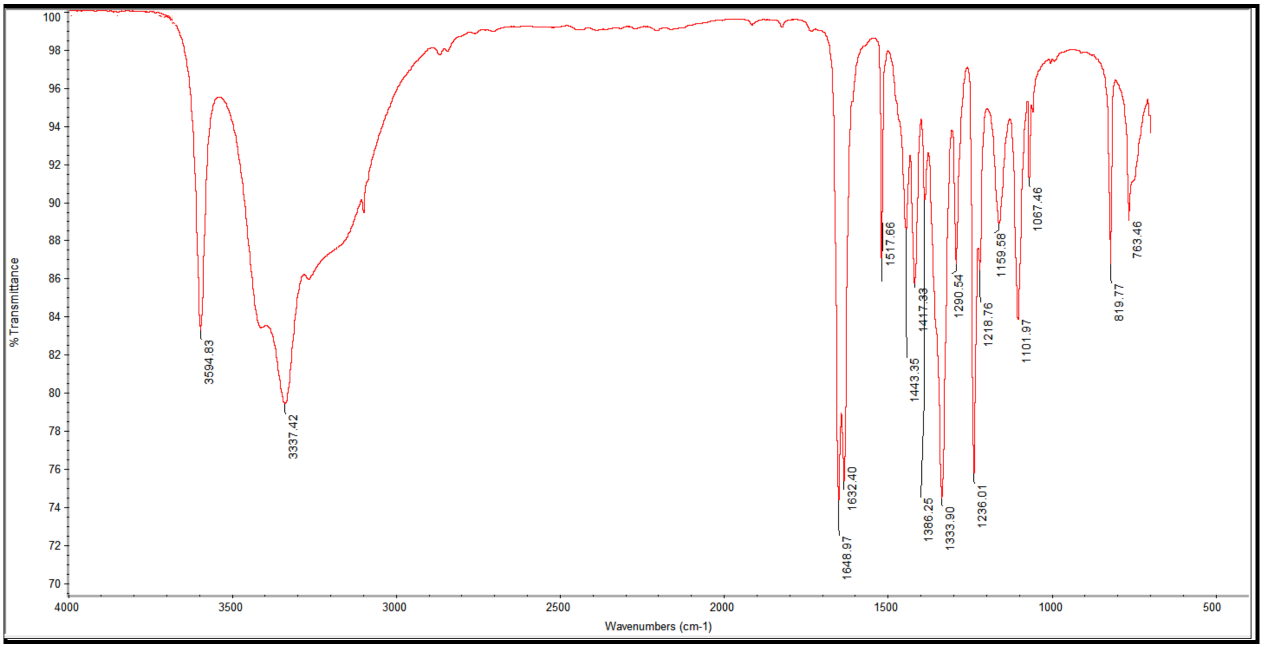

Fig. S16. FTIR spectra for **IMA4**

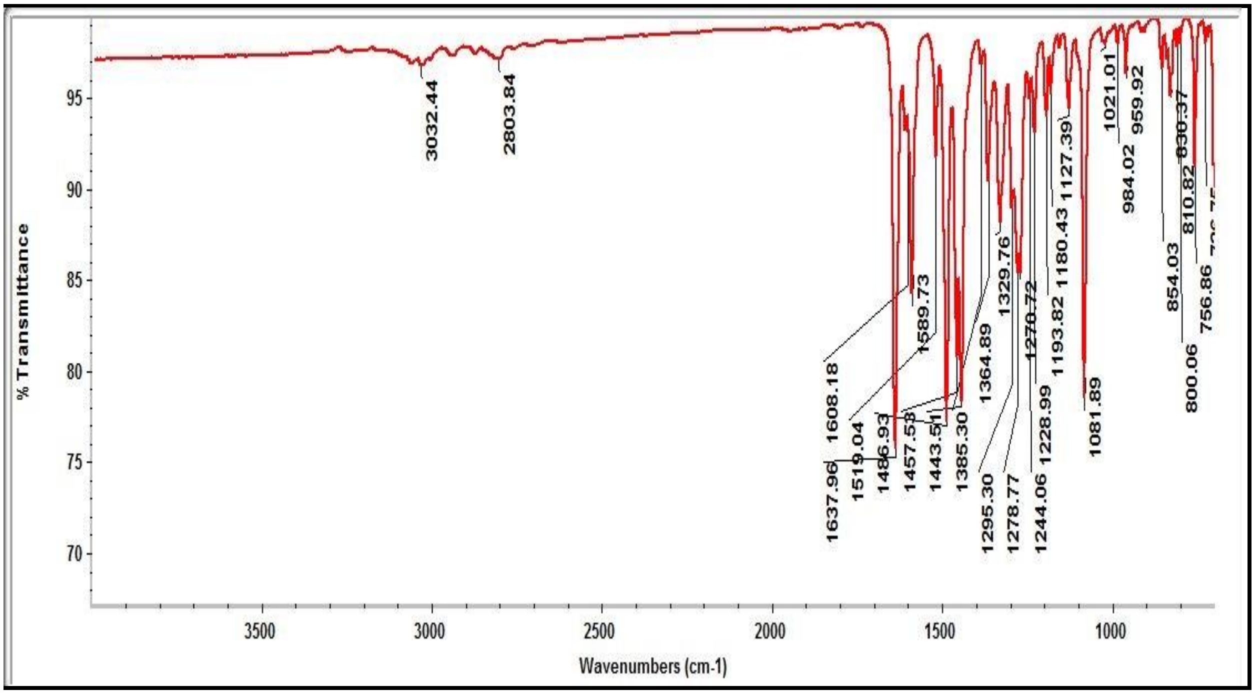

Fig. S17. FTIR spectra for **9**

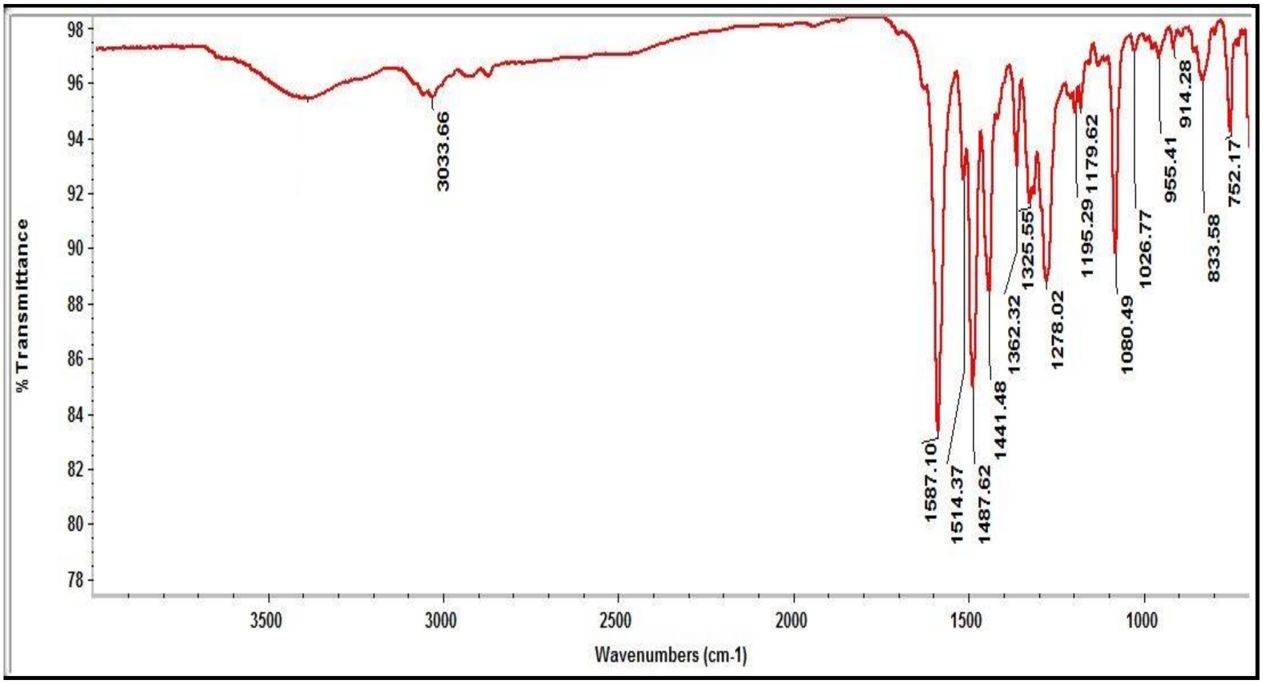

Fig. S18. FTIR spectra for **10**

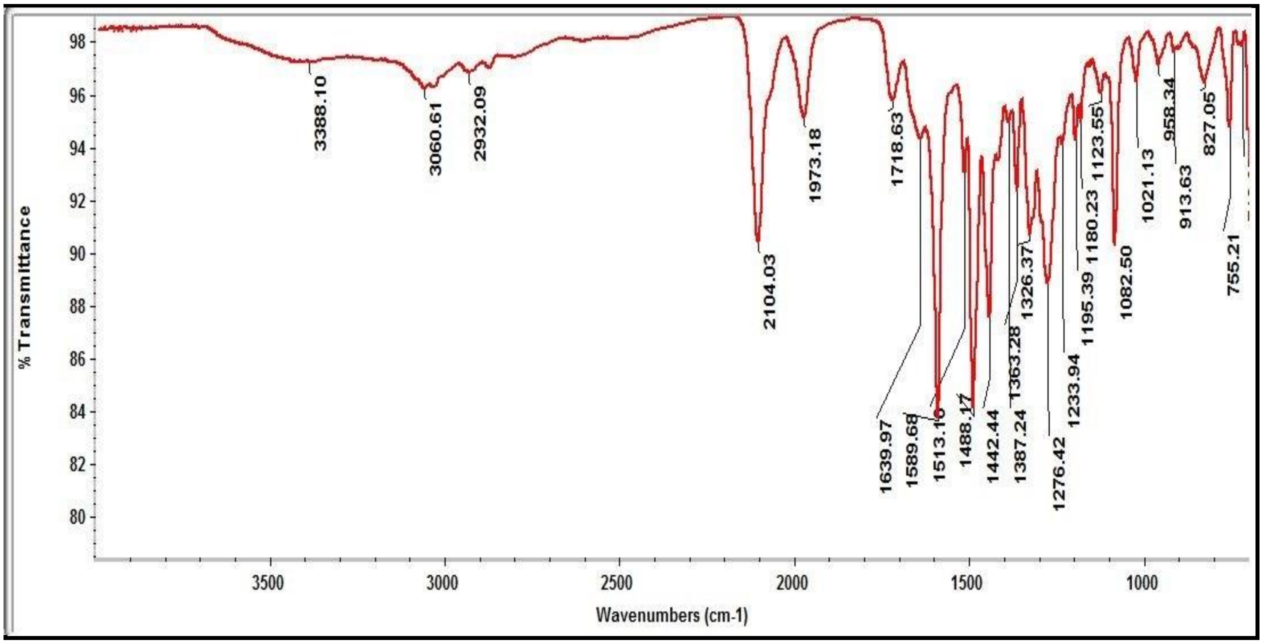

Fig. S19. FTIR spectra for **IMA5**

4.3.^{1}H-NMR spectra

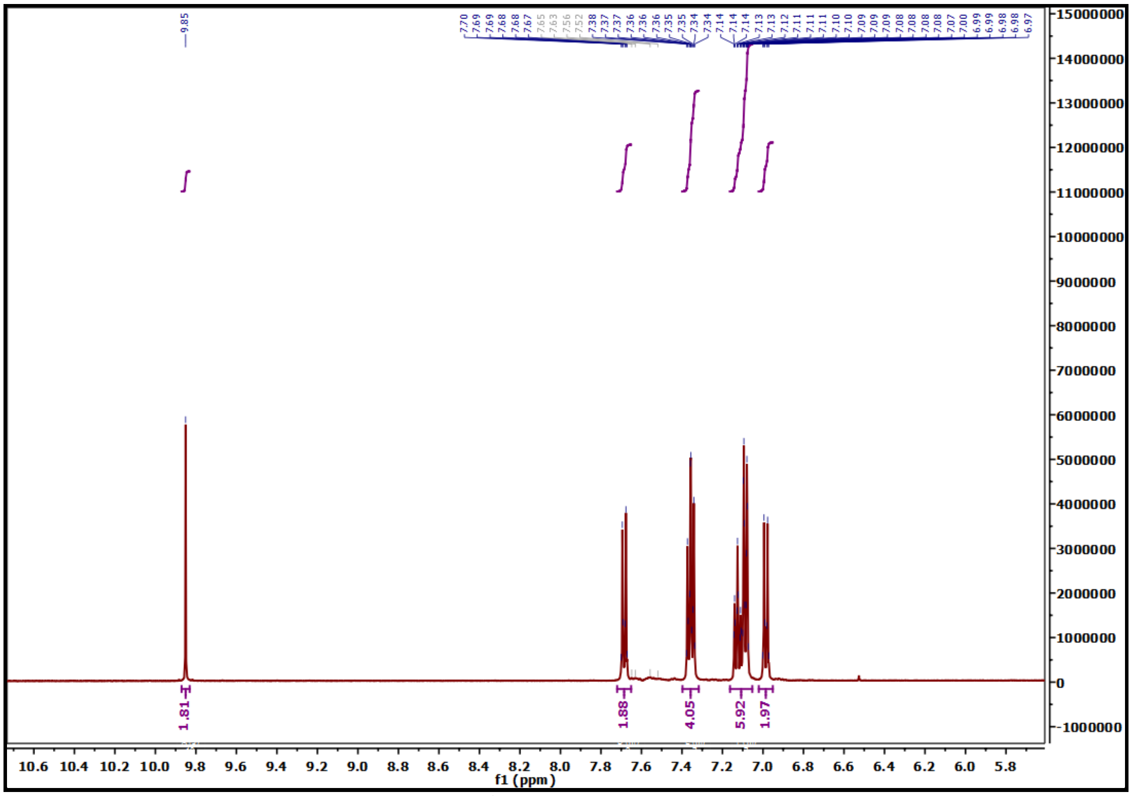

Fig. S20. ^{1}H-NMR spectra for **2**

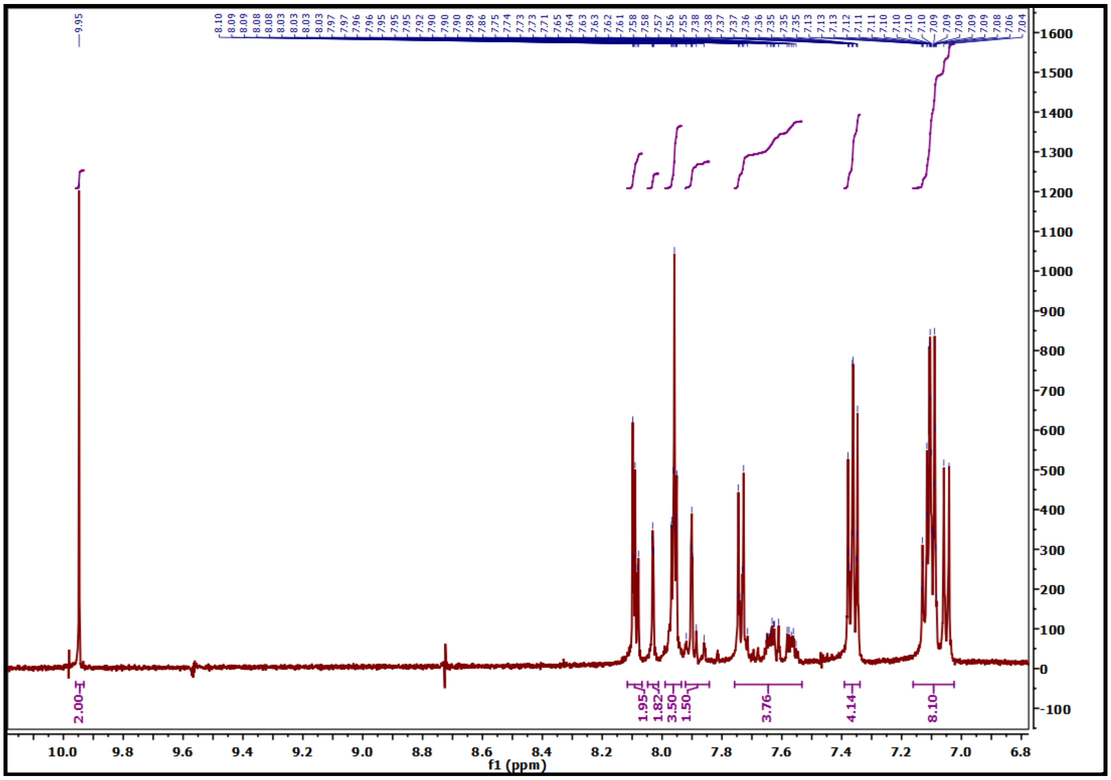

Fig. S21. ^{1}H-NMR spectra for **IMA1**

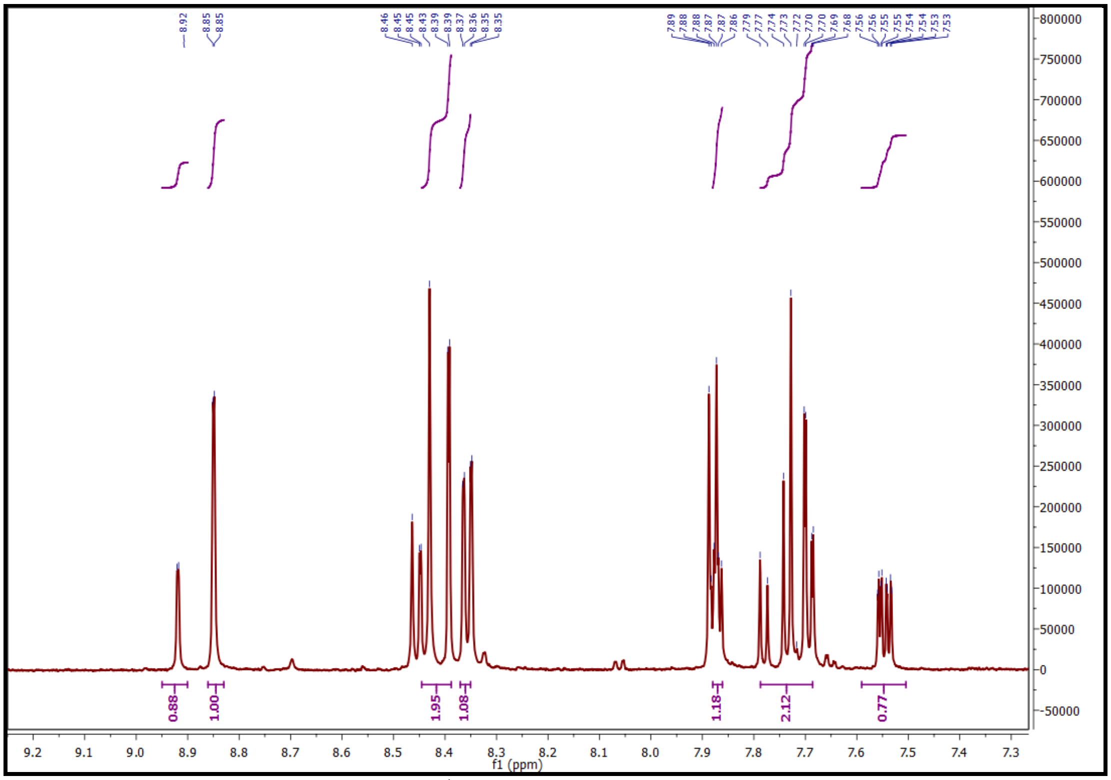

Fig. S22. ^{1}H-NMR spectra for **IMA2**

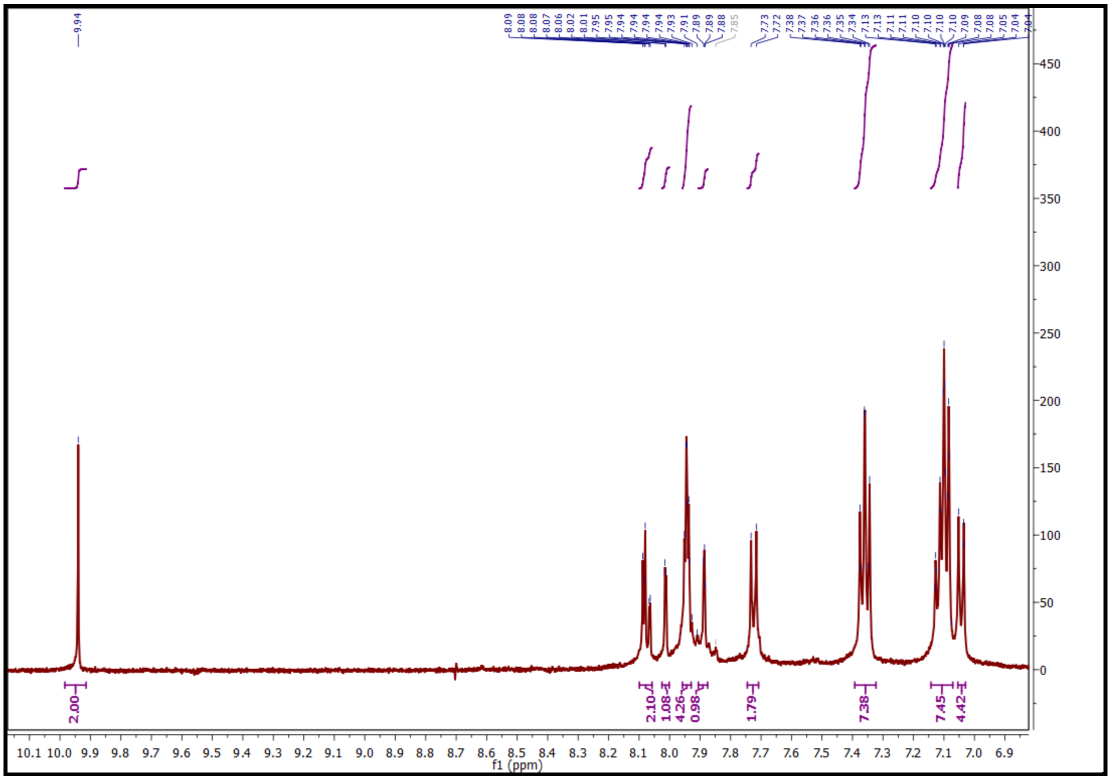

Fig. S23. ^{1}H-NMR spectra for **IMA3**

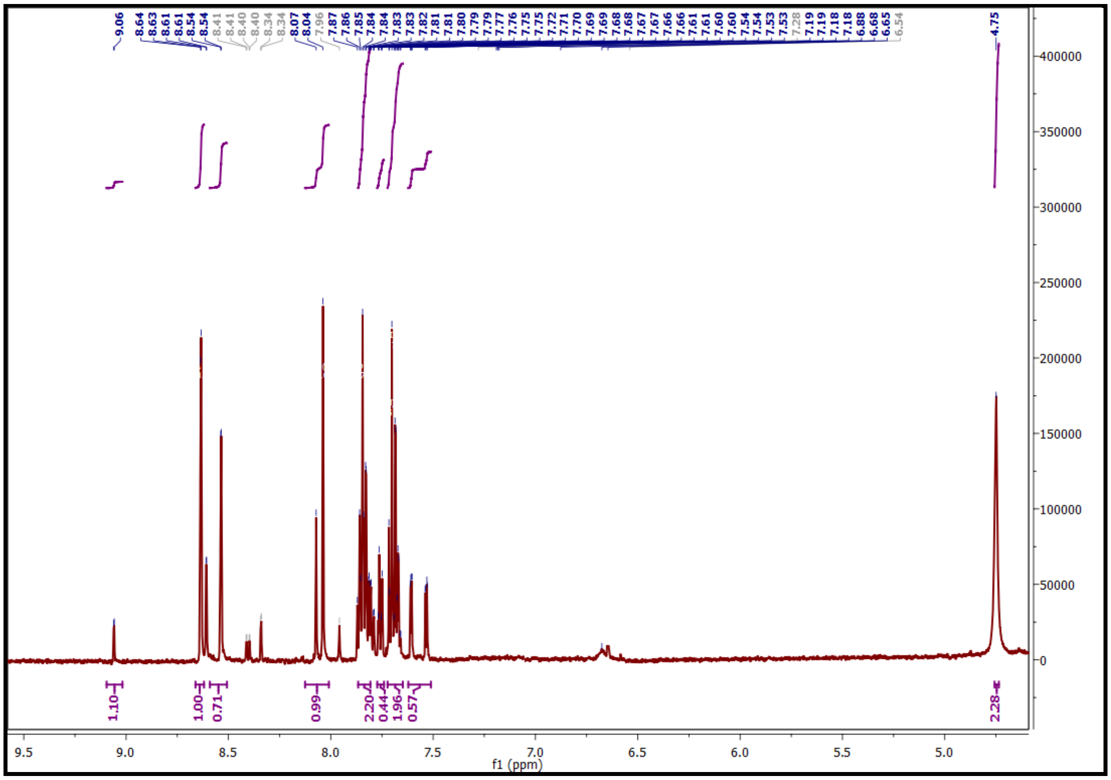

Fig. S24. ^{1}H-NMR spectra for **IMA4**

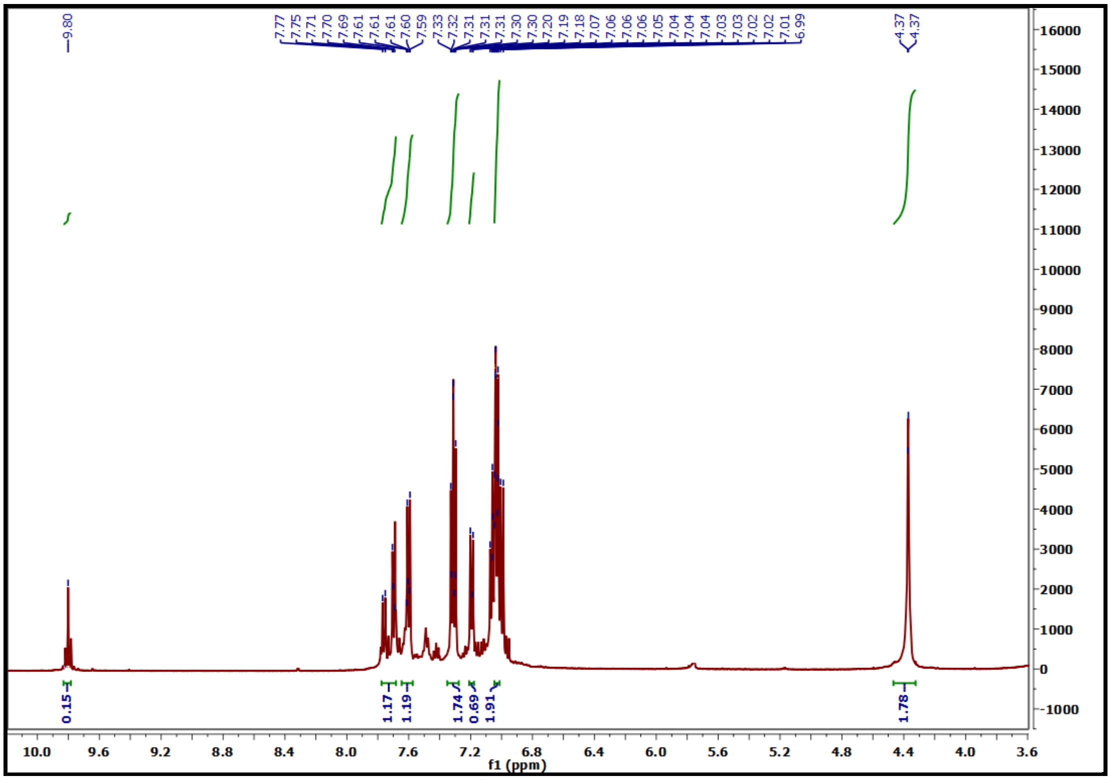

Fig. S25. ^{1}H-NMR spectra for **9**

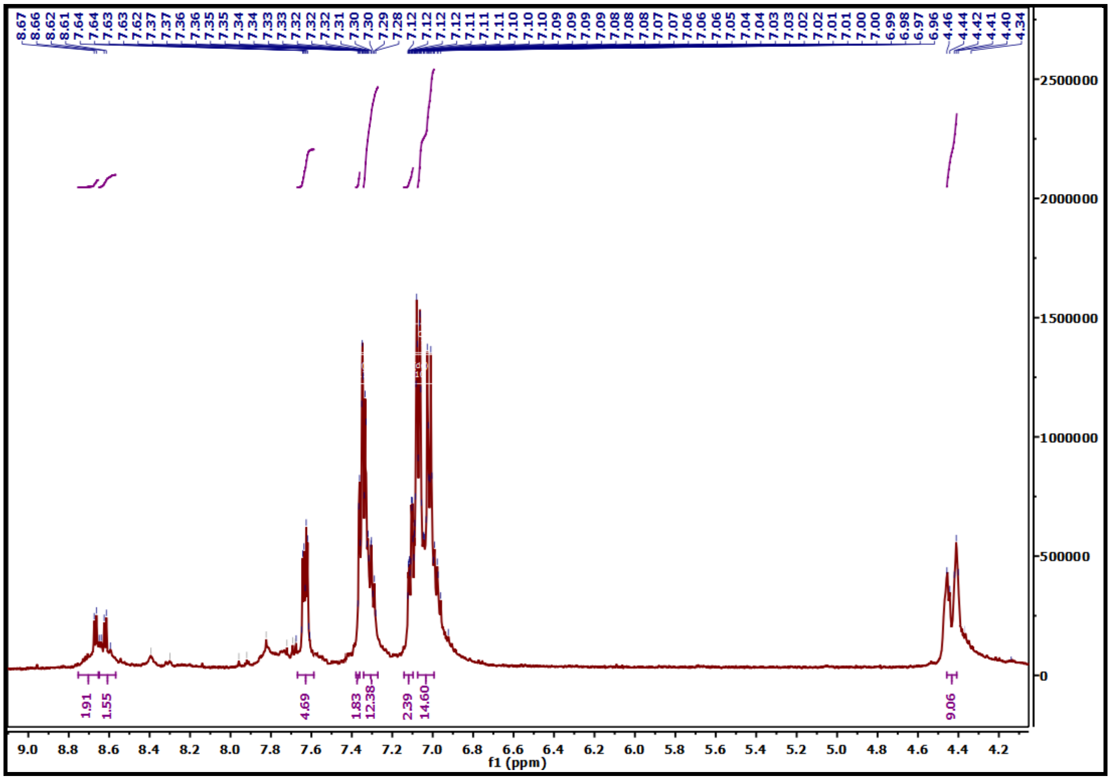

Fig. S26. ^{1}H-NMR spectra for **10**

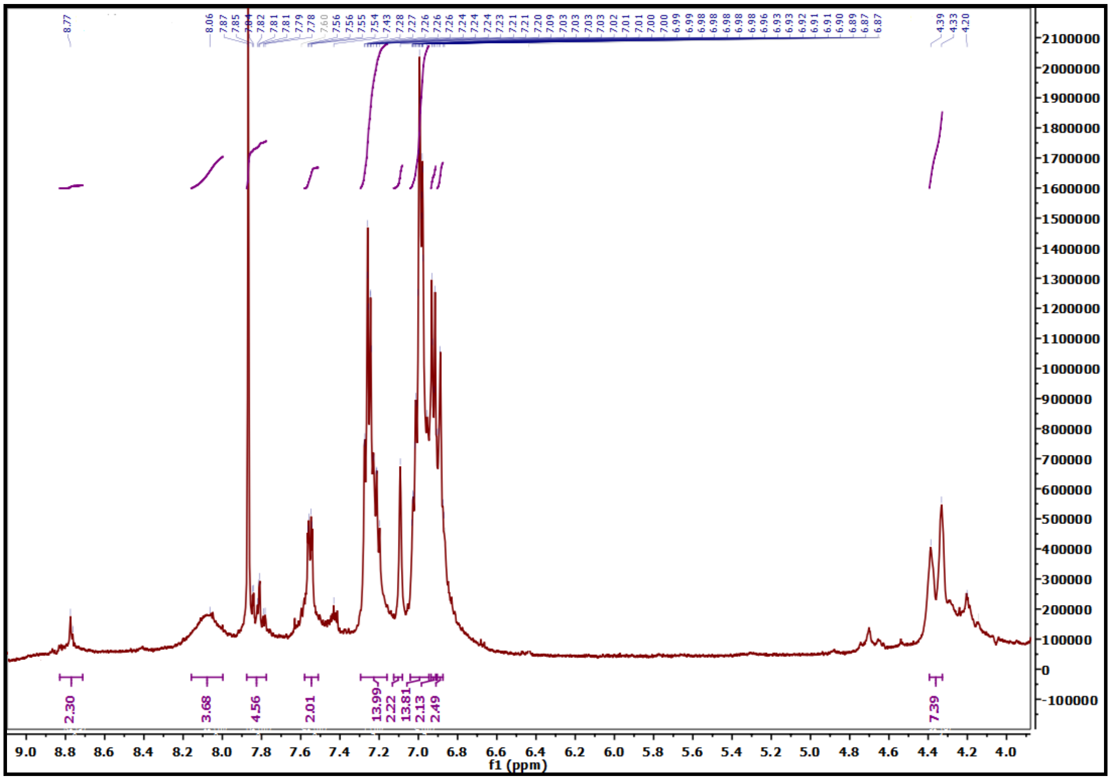

Fig. S27. ^{1}H-NMR spectra for **IMA5**
